# Chicken CSF2 and IL-4-, and CSF2-dependent bone marrow cultures differentiate into macrophages over time

**DOI:** 10.3389/fimmu.2022.1064084

**Published:** 2022-12-21

**Authors:** Dominika Borowska, Samantha Sives, Lonneke Vervelde, Kate M. Sutton

**Affiliations:** The Division of Infection and Immunity, The Roslin Institute and Royal (Dick) School of Veterinary Studies, University of Edinburgh, Edinburgh, United Kingdom

**Keywords:** bone marrow cell cultures, chicken, dendritic cell, macrophage, transcriptome

## Abstract

Chicken bone marrow-derived macrophages (BMMΦ) and dendritic cells (BMDC) are utilized as models to study the mononuclear phagocytic system (MPS). A widely used method to generate macrophages and DC *in vitro* is to culture bone marrow cells in the presence of colony-stimulating factor-1 (CSF1) to differentiate BMMΦ and granulocyte-macrophage-CSF (GM-CSF, CSF2) and interleukin-4 (IL-4) to differentiate BMDC, while CSF2 alone can lead to the development of granulocyte-macrophage-CSF-derived DC (GMDC). However, in chickens, the MPS cell lineages and their functions represented by these cultures are poorly understood. Here, we decipher the phenotypical, functional and transcriptional differences between chicken BMMΦ and BMDC along with examining differences in DC cultures grown in the absence of IL-4 on days 2, 4, 6 and 8 of culture. BMMΦ cultures develop into a morphologically homogenous cell population in contrast to the BMDC and GMDC cultures, which produce morphologically heterogeneous cell cultures. At a phenotypical level, all cultures contained similar cell percentages and expression levels of MHCII, CD11c and *CSF1R*-transgene, whilst MRC1L-B expression decreased over time in BMMΦ. All cultures were efficiently able to uptake 0.5 µm beads, but poorly phagocytosed 1 µm beads. Little difference was observed in the kinetics of phagosomal acidification across the cultures on each day of analysis. Temporal transcriptomic analysis indicated that all cultures expressed high levels of *CSF3R*, *MERTK*, *SEPP1*, *SPI1* and *TLR4*, genes associated with macrophages in mammals. In contrast, low levels of *FLT3*, *XCR1* and *CAMD1*, genes associated with DC, were expressed at day 2 in BMDC and GMDC after which expression levels decreased. Collectively, chicken CSF2 + IL-4- and CSF2-dependent BM cultures represent cells of the macrophage lineage rather than inducing conventional DC.

## Introduction

Cells of the mononuclear phagocyte system (MPS) differentiate from pluripotent stem cells in response to hemopoietins, such as colony-stimulating factor (CSF1), granulocyte-macrophage-CSF (GM-CSF, CSF2) or fms-like tyrosine kinase receptor 3 ligand (FLT3L). Macrophages are a heterogeneous cell population, involved in host defense against pathogens, using phagocytosis to engulf and dispose of foreign particles, a process also utilized to regulate tissue and cell damage to control tissue repair and maintain homeostasis. Macrophages acquire tissue-specific characteristics, and differ in terms of ontogeny, phenotype, and functionality despite their shared cellular origin (1). Dendritic cells (DC) are made up of distinct sub-groups; conventional DC, 1 and 2 (cDC1 & 2) and plasmacytoid DC (pDC). Conventional DC link the innate and adaptive arms of the immune response through their ability to present antigens to naïve T cells (2) while pDC have a key role in host defense against virus infection (3).

Both macrophages and DC are scattered throughout the body, it is generally difficult to isolate sufficient numbers for comprehensive studies without using enzymatic extraction steps that may detrimentally affect cell surface marker expression. Therefore, the majority of research either relies on isolating circulating monocytes to generate monocyte-derived macrophages or bone marrow (BM) cells cultured in the presence of specific cytokines to promote macrophage and DC differentiation for the production of each cell lineage on a large scale. In mouse and human, BM cells can differentiate into macrophages using CSF1 (4), while murine BMDC have been established using CSF2 (2). Soon after, the addition of interleukin 4 (IL-4) with CSF2 was shown to be required for the generation of human BM- and monocyte-derived DC (5). Based on phenotype and morphology, human BMDC (CSF2 + IL-4) represent immature DC while the addition of IL-4 to murine CSF2 treated BM cultures induces DC maturation (5). Generally in mammals, DC maturation is described based on the expression levels of major histocompatibility complex class II (MHCII), CD11c and co-stimulatory molecules, CD40 and CD86 (6). Functionally, apart from phagocytosis, DC have an additional attribute, their ability to cross-present antigens to naïve T cells (7). However, studies are increasingly showing that macrophages have the capacity to cross-present but whether this is a requirement for primary or memory T cell responses is still to be elucidated (8, 9).

Both BMMΦ and BMDC have been generated in veterinary species such as pigs, ruminants, dogs and cats, similar to the original protocol published for human and mouse (10–13). Studies have reported that classical methods for macrophage and DC generation produces a heterogeneous population of cells. For example, murine BMDC cultured with CSF2 alone generated both macrophages and neutrophils (14) while another study demonstrated the presence of macrophages and DC in CSF2 treated cells (6, 15). Similarly, when CSF2 is replaced by FLT3L in murine and porcine BM cultures, the cells resembled a bona fide cDC lineage (11, 16, 17).

Transcriptomic analysis of MPS cells derived from distinct tissues led to defined core macrophage and DC signature genes (1, 18–20). These core gene sets have been utilized to examine DC and macrophage lineages in a number of chicken tissues (21–24). Recently, novel reagents to chicken FLT3 and XCR1, cDC1 markers, demonstrated that chicken BMDC lack the expression of these proteins (25). Studies have shown macrophage development in chicken CSF1-treated BM cells (22, 26, 27). However, few studies have defined the lineage of CSF2 + IL-4 or CSF-2 treated chicken BM cells and functional analysis of CSF1 and CSF2 + IL-4 treated BM are lacking. The aim of our study was to gain a detailed understanding of the developmental kinetics and transcriptome dynamics of BM-derived cultures, based on morphology, phenotype, function and transcriptome, for validation of *in vitro* models of macrophages and DC. The present study uniquely derived BM cells from individual birds and followed their growth in the presence of both CSF2 + IL-4 (BMDC), CSF2 alone (GMDC) or CSF1 (BMMΦ) and their simultaneous characterization at days 2, 4, 6 and 8 in culture to achieve an in-depth kinetic comparison. In addition, transcriptome dynamics of BM-derived cultures gave a comprehensive characterization of cells throughout the culture period. Conclusively, chicken CSF2 + IL-4 and CSF2-dependent BM cultures represented cells of the macrophage lineage rather than inducing conventional DC.

## Materials and methods

### Chickens and ethical statement

*CSF1R*-reporter transgenic chickens and wild type Hy-Line brown chickens were used at 4-6 weeks of age and provided by the National Avian Research Facility (NARF), The Roslin Institute, Edinburgh, UK. The unvaccinated chickens were reared in floor pens and maintained under conventional conditions with water and feed *ad libitum.* Animals were housed in premises licensed under a UK Home Office Establishment License in full compliance with the requirements of the Animals (Scientific Procedures) Act 1986. Breeding of transgenic chickens was carried out under the authority of Project License PPL70/8940 with the consent of The Roslin Institute Animal Welfare and Ethical Review Board.

### Bone marrow cell isolation and cell culture maintenance

Birds were humanely culled by cervical dislocation in accordance with Schedule 1 of the Animals (Scientific Procedures) Act 1986, and femurs and tibias were removed and stored in PBS on ice until use. Both ends of the bones were cut and the medulla was flushed with 10 mL of PBS (pH 7.4, Ca^2+^ and MgCl^2+^ free, used throughout the study) using 21G needle and 10 mL syringe. The bone marrow was pressed through a 70 µm strainer and cells were pelleted at 400 x *g* for 10 min at room temperature (RT), resuspended in PBS and gradient purified (Histopaque 1.077; Sigma-Aldrich, Gillingham, UK) in 1:1 ratio for 20 min at 400 x *g* with no brakes. The interface and cells above were retrieved and washed twice with complete media (RPMI with 10% heat inactivated fetal bovine serum (FBS, GIBCO), L-glutamine (2 mM), penicillin (20 U/mL) and streptomycin (20 µg/mL), ThermoFisher Scientific (TFS). Live cells were counted using Trypan Blue exclusion (Corning, USA). All BM cells were cultured in complete RPMI supplemented with 10 ng/mL recombinant chicken IL-4 and CSF2 (Kingfisher Biotech Inc., USA) for BMDC, 10 ng/mL CSF2 for GMDC and 200 ng/mL of CSF1 for BMMΦ [produced in house (26)]. The optimal concentration of cytokines was determined based on the observed morphology of cells as described (26, 28). Cells were incubated at 41˚C, 5% CO_2_, conditions used throughout the study. For phenotypical and functional analysis, BM were isolated from six individual chickens over two independent experimental days (n=3 per experiment) and cultured independently with CSF1, CSF2 + IL-4 or CSF2 for 2, 4, 6 and 8 days of culture.

On days 3 and 6 of culture, two-thirds of the cell culture media was removed (dead and non-adherent cells) and replaced with fresh complete RPMI supplemented with the appropriate cytokines. For phenotypical analysis using flow cytometry, 10^6^ cells/mL in 3 mL were seeded in 6-well plates. Prior to harvesting, cells were gently washed with PBS and detached from the wells by the addition of 1 mL of TrypLE Express (Life Technologies, UK) and incubated at 41˚C for 10 min. The cells were further dislodged from the wells by vigorous pipetting and the enzymatic reaction was quenched by the addition of complete RPMI. The cells were centrifuged at 400 x *g* for 5 min at RT, resuspended in complete RPMI, and counted.

### Flow cytometric analysis

On days 2, 4, 6 and 8 of culture, cells were harvested as outlined above, counted and adjusted to ~10^7^cells/mL and stained as previously described (23). For multi-color flow cytometric analysis, primary antibodies were conjugated with PerCP-Cy5.5, PE-Cy7 or APC using Lightning-Link Antibody Labelling Kit (Novus Biologicals, USA), following the manufacturer’s instructions. All antibodies were titrated prior to use with details outlined in **Table 1**. Prior to phenotypical, phagocytosis and acidification analysis, cells were treated for 5 min with Sytox™ Blue LIVE/DEAD stain (TFS) and a minimum of 10K live, single cells were collected for each sample. Compensation was achieved using BD mouse IgGκ Compensation beads according to the manufacturer’s instructions (TFS). All flow cytometry-based experiments were performed using BD LSR Fortessa™ with 4 lasers and 16 filters (BD Biosciences, UK). Analysis was carried out using FlowJo (TreeStar v10). Fluorescence intensities are displayed on ‘logical’ scales showing negative value. Fluorescence minus one controls (FMO) and wild type, non-transgenic animals were used to apply gates.

**Table 1:** Primary antibodies for the phenotypical analysis of BM-derived cultures.

Antibody	Isotype	Clone	Antigen	Supplier	Working concentration
Mouse Anti-chicken Monocyte/Macrophage-PE	IgG1	KUL01	MRC1L-B	SB^1^	0.125 µg/ml
Mouse Anti-chicken CD45-SPRD	IgM	LT40	CD45	SB	0.5 µg/ml
Mouse anti chicken MHC II	IgG1	2G11	MHC II β chain	SB	1 µg/ml
Mouse anti-chicken CD40	IgG2a	IG8	CD40	RI^2^	2 µg/ml
Mouse anti-chicken CD11c	IgG1	8F2	Putative CD11c	LMU^3^	1 µg/ml

^1^Southern Biotech; ^2^Immunological Toolbox, The Roslin Institute; ^3^gift from Dr. S. Härtle, LMU, Germany.

### Phagocytosis and acidification assays

The BM cell cultures were harvested on days 2, 4, 6 and 8 and reseeded at 10^6^ cells/well in two identical 96-well plates; 41°C phagocytosis assay and 4°C controls. At 2 h post-harvest, 10^7^ (10:1 bead to cell ratio) 0.5 µm or 1.0 µm red fluorescent FluoSpheres™ Carboxylate-Modified Microspheres (TFS) were added to the cells for 2 h and incubated at 41°C or on ice to determine non-specific uptake and application of flow cytometric gates. Phagocytosis was inhibited by the addition of ice-cold PBS and cells were washed twice with ice-cold PBS before harvesting using TrypLE Express as outlined previously.

Under the same conditions, acidification of intracellular vesicles was measured using pHrodo^®^ Red Zymosan BioParticles^®^ (Invitrogen, UK). The bioparticles were added at a 10:1 particle to cell ratio for 2 h at 41°C. To determine non-specific bioparticle uptake and application of flow cytometric gates, cells were treated with the actin polymerization inhibitor, cytochalasin D (CytoD, 20 µM, Cayman Chemical, USA) for 15 min prior to treatment with bioparticles. Cells were washed twice with PBS and detached using TrypLE Express as outlined previously. Phagocytosis and acidification was measured using flow cytometry.

Real-time analysis of acidification across the BM cell cultures on days 2, 4, 6 and 8 of culture were measured using CLARIOstar plate reader in a controlled atmosphere, allowing for longitudinal analysis of the fluorescence emitted by the acidified pHrodo Red Zymosan BioParticles. Cells were harvested and seeded at 10^5^ cells per well in black 96-well clear bottom plates (Greiner Bio-One Inc., USA) and incubated for a further 4 h at 41°C, 5% CO_2_ before addition of the BioParticles at 10:1 particle to cell ratio for 18 h. Control cells were treated with CytoD as outlined above. The intensity was measured at 15 min post-treatment followed by every hour for 18 h. The raw data were normalized to control cells treated with CytoD.

### Measurement of nitric oxide (NO) production

Nitrite (NO_2_) concentration, as an index of nitric oxide (NO) production, was measured at days 2, 4, 6 and 8 of culture. The BM cells cultures were reseeded at 10^6^ cells/well in duplicate wells on 12-well plates and treated with 10 ng/mL lipopolysaccharide (LPS) *E. coli* O55:B5 (Sigma-Aldrich) for 48 h after which the supernatant was collected and stored at -20°C until use. Griess Reagent Kit was used to measure NO production in 96-well flat bottom plate following the manufacturer’s instructions (Invitrogen, UK). To calculate NO production, dilutions of sodium nitrite, ranging from 0.78 to 100 μM, were measured to generate a standard curve. Absorbance was measured at 570 nm using a SpectraMax plate reader.

### Total RNA extraction and RNA-sequencing

Bone marrow cultures derived from three individual chickens were independently cultured with CSF1, CSF2 + IL-4 or CSF2 on 6-well plates. On days 2, 4, 6 and 8 of culture, floating cells were discarded and cells were gently washed with PBS to prevent dislodging of the semi-adherent cells. Cells were lysed with RLT buffer (QIAGEN, UK) supplemented with β-mercaptoethanol (10 µm, TFS). Total RNA was isolated using the RNEasy mini kit (QIAGEN) following the manufacturer’s instructions. RNA quantity and quality were assessed using Agilent RNA ScreenTape using TapeStation 2200 (Agilent, UK). All samples had an RNA integrity index > 9.5. Illumina TruSeq stranded mRNA-seq libraries were generated for the thirty-six samples and sequenced on NovaSeq by Edinburgh Genomics UK yielding at least 26-123.9M mapped read pairs per sample.

### RNA-seq analysis

RNA-seq reads were trimmed for quality at the 3’ end using a quality threshold of 30 and for adapter sequences of the TruSeq stranded mRNA kit (AGATCGGAAGAGC) with a minimum length of 50 bp using Cutadapt1 (version cutadapt-1.9.dev2). Transcripts were mapped to the Gallus_gallus-5.0 genome using STAR2 (version 2.5.2b). Raw counts for each annotated gene were obtained using the featureCounts3 software [version 1.5.1). Principal Components Analysis (PCA) was analyzed using normalized and filtered data to explore observed patterns with respect to experimental factors. Statistical assessment of differential expression analyzed using quasi-likelihood (QL) F-test using a false-discovery rate (FDR) < 0.05 and log2 fold-change (FC) > 2. The heat maps of core signature genes expression in the cultures were created in GraphPad based on the fragment per kilobase per million reads mapped (FPKM) values and presented as log10 FPKM with addition of 1 to avoid visual bias from genes with FPKM values <1.

### Network gene analysis

Gene-to-gene network analysis was performed in Graphia [https://graphia.app//] (29). Pairwise Pearson correlations (r > 0.95) were calculated between all the cultures from day 4 to day 8 to create a matrix of correlations for each pair of genes. Network graphs were created by connecting nodes (genes) with edges (connectivity based on correlation above the defined threshold) and its local structure defined by applying the Louvain clustering algorithm at an inflation value (cluster granularity) of 0.650. Over-representation of gene ontology (GO) terms were derived from PANTHER (version 15.0) (http://pantherdb.org/tools/ *Gallus gallus* as a reference organism), statistical overrepresentation test using Fisher’s Exact with FDR multiple test correction, DAVID Bioinformatics Resources (version 6.8, https://david.ncifcrf.gov/) and gProfiler (https://biit.cs.ut.ee/gprofiler/gost, Homo sapiens as a reference organism).

### Statistical analysis

Statistical analysis was performed using GraphPad Prism 8.00 (GraphPad, San Diego, USA). All data was analyzed for normality. All data was analyzed by two way Anova test adjusted for *post-hoc* analysis. The probability level for significance was taken as p ≤ 0.05.

## Results

### CSF1 induces a morphologically homogenous cell culture in contrast to CSF2 + IL-4 treated cultures

**Figure 1:**
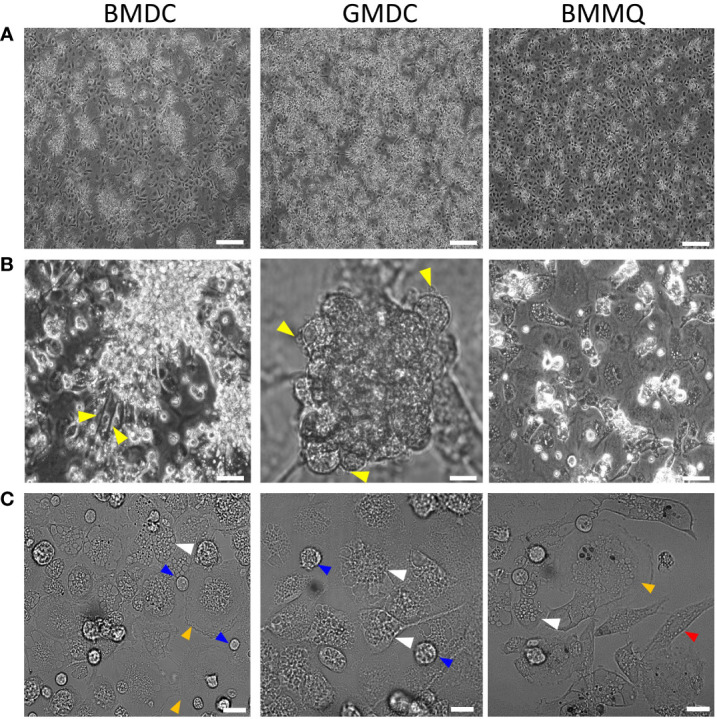
CSF1 induces a morphologically homogenous BM cell cultures in contrast to BM treated with CSF2 + IL-4 or CSF2 alone. **(A)** Representative DIC images of chicken BM cells treated with CSF2 + IL-4 (BMDC), CSF2 (GMDC) or CSF1 (BMMΦ) on day 4 of culture. **(B)** Yellow triangles indicate veiled cells **(C)** white triangles indicate cells with numerous intracellular vacuoles, blue triangles shows small round cells and orange triangles show large cells and red triangles show elongated cells. Images of three independent BM cultures derived from 5-week-old chickens. Scale bar represents 100 µm in A and 50 µm in B and C.

BM cells from 4- to 6-week-old chickens were cultured for up to 8 days in the presence of different cytokines; CSF1 to generate BMMΦ, CSF2 to generate GMDC, and a combination of CSF2 + IL-4 to generate BMDC. Firstly, we examined the effect of cytokines on cellular morphology at day 4 of culture (**Figure 1**). Semi-adherent clusters with underlying adherent cells, with a “fried egg” morphology, were observed in both BMDC and GMDC cultures (**Figure 1A**). In contrast, clusters of cells were rarely observed in the CSF1 treated cultures. Upon closer analysis, BMMΦ cultures were more homogenous and consisted predominantly of cells with a “fried egg” morphology (**Figures 1A, B**). Clusters in the BMDC and GMDC cell cultures consisted of numerous small round cells with short protrusions (**Figure 1B**). Irrespective of treatment, adherent cells presented with numerous intracellular vacuoles and both large round, elongated cells were observed in each culture. There was little difference in the cellular morphology from day 4 onwards (data not shown). Without the addition of cytokines, the presence of few floating cells and very little cell adherence was observed by day 4 (**Figure S1**). As few cells survived after 4 days of culture, these non-treated cells were omitted from further downstream studies.

### BMDC, GMDC and BMMΦ develop similar MHCII^+^ and CD11c^+^ subpopulations

BMDC, GMDC and BMMΦ cultures were primarily analyzed based on flow cytometric side and forward scatter profiles (**Figure S2**). Side scatter analysis did not show any differences in granularity between cultures and the forward scatter patterns indicated that BMMΦ were slightly larger in size at day 2 of culture compared to BMDC and GMDC cultures. From day 4 of culture, all cell cultures were characterized by the same pattern, based on size and granularity (**Figure S2**).

**Figure 2:**
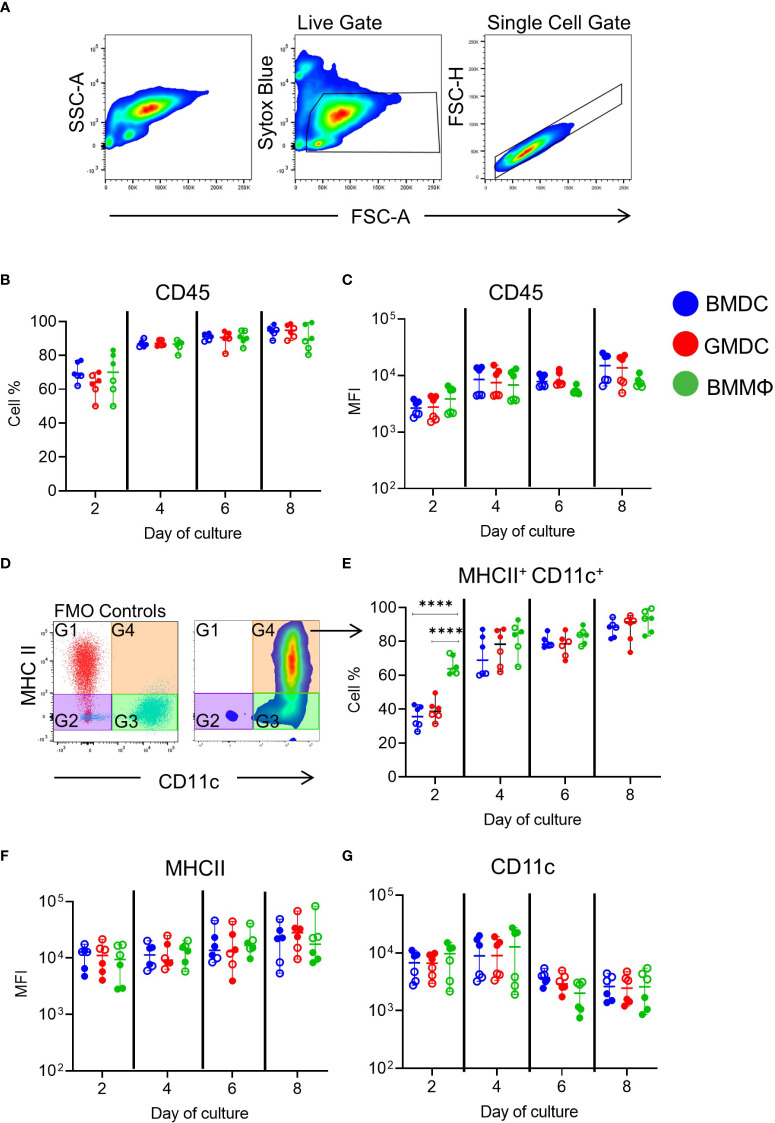
Phenotypic analysis of chicken BMDC, GMDC and BMMΦ on days 2, 4, 6 and 8 of culture. **(A)** Representative flow plots of SSC-A and FSC-A and live and single cell gating strategy applied to all cultures at each day of analysis. **(B)** The percentage of CD45^+^ cells and **(C)** median fluorescence intensity (MFI) levels of CD45 expression live, single cells in BMDC (blue), GMDC (red) and BMMΦ (green) on days 2, 4, 6 and 8. **(D)** Dot plots representing FMO-1 control gating strategy on live, single cells for the analysis of MHCII^+^ CD11c^+^ (G3) cell percentages and **(E)** MFI of **(F)** MHCII and **(G)** CD11c expression levels in G4 across each culture on days 2, 4, 6 and 8. Data represents the median (± range). From each bird (n=6), BMDC, GMDC and BMMΦ were generated. Data represents two independent experiments (n=3 per experiment, open & closed circles). Statistical significant differences between cultures on day of analysis are indicated by ****p<0.001.

To investigate phenotypic differences between cultures, expression of chicken MPS cell surface markers was analyzed over time using flow cytometry. Due to possible differences in individual culture reactivity, we conducted two independent experiments with each consisting of three individual chickens for all phenotypical and functional analysis (**Figure 2**, open & closed circles). Phenotypical analysis was carried out by applying a live, single cell gates to each culture and analyzing the expression of specific markers (**Figure 2A**). Firstly the percentage of CD45^+^ cells in the live, single gate was analyzed (**Figure 2B**) and its median fluorescence intensity (MFI; **Figure 2C**), showing an increase over time but did not differ significantly between the cultures.

Next, cells expressing both MHC class II (MHCII) and CD11c, using the putative anti-chicken CD11c monoclonal, 8F2, were analyzed across each culture over time to resemble the approach used in mammals. Using FMO controls, four gates were applied to the live, single cells in each culture (**Figure 2D**). The percentage of MHCII^+^ CD11c^+^ cells within each culture increased over time and reached ~80-90% by day 8 (**Figure 2E**; **Figure S3A**). At day 2, a significantly higher percentage of MHCII^+^ CD11c^+^ cells were observed in BMMΦ compared to BMDC and GMDC cultures, while from day 4 onwards all cultures had similar percentages of MHCII^+^ CD11c^+^ cells (**Figure 2E**; **Figure S3**). The MFI of MHCII and CD11c did not significantly differ between the cultures (**Figures 2F, G**). Each culture contained a major MHCII^+^ CD11c^+^ subpopulation.

### All MHCII^+^ and CD11c^+^ cells express similar levels of chicken macrophage markers

**Figure 3:**
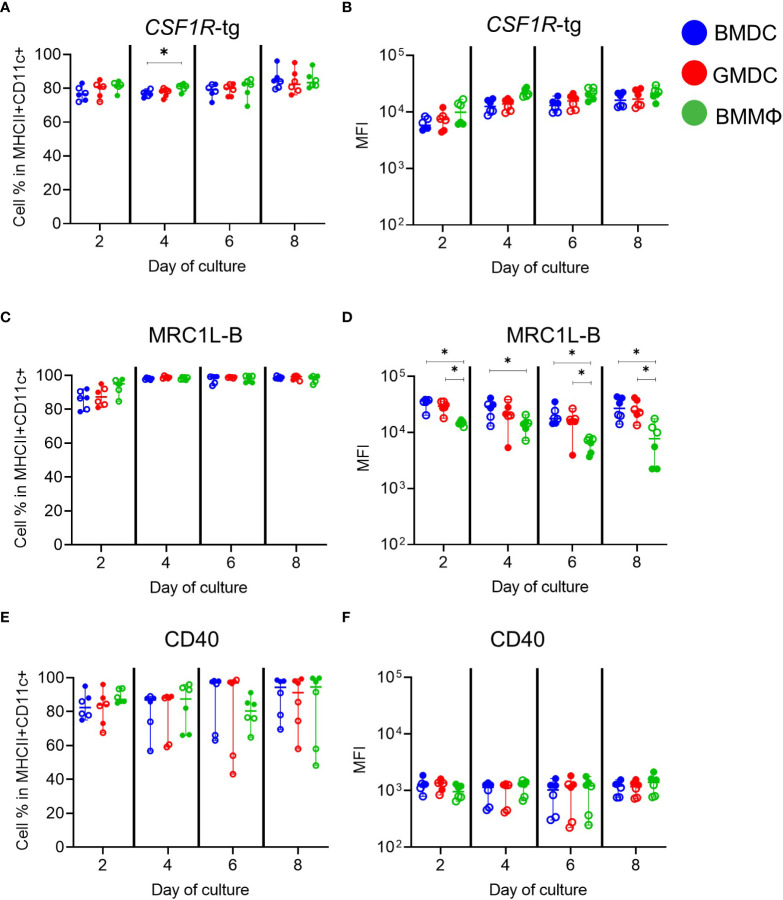
Phenotypic analysis of MHCII^+^ CD11c^+^ subpopulations in chicken BMDC, GMDC and BMMΦ cultures on days 2, 4, 6 and 8. The cell percentages and MFI of **(A, B)** CSF1R-tg, **(C, D)** MRC1L-B and **(E, F)** CD40 in the MHCII^+^ CD11c^+^ subpopulations in BMDC (blue), GMDC (red) and BMMΦ (green) on days 2, 4, 6 and 8. Data represents the median (± range). From each bird (n=6), BMDC, GMDC and BMMΦ were generated. Data represents two independent experiments (n=3 per experiment, open & closed circles). Statistical significant differences analyzed between cultures on day of analysis are indicated by *p<0.05.

Next, further phenotypical analysis focused on the MHCII^+^ CD11c^+^ subpopulation in each culture (**Figure 3**). We used *CSF1R*-reporter transgenic chickens, which express a transgene under control of the *CSF1R* promoter and FIRE enhancer, essentially labelling most cells of the MPS (30). Approximately 70-80% of the MHCII^+^ CD11c^+^ subpopulation expressed the *CSF1R*-transgene (*CSF1R*-tg; **Figure 3A**). There was no significant difference observed in the MFI of *CSF1R*-tg expression between the cells across the different days of culture (**Figure 3B**). Expression of MRC1L-B (recognized by the MoAb KUL01) in the MHCII^+^ CD11c^+^ subpopulations increased from day 2 to day 4 of culture reaching 95-100%, and plateaued for the duration of the culture period (**Figure 3C**). This also demonstrates that all cells express MRC1L-B but ~10-20% lack *CSF1R*-tg expression (**Figure S3B**). The MFI of MRC1L-B expression was significantly lower on the BMMΦ on all days compared to BMDC (**Figure 3D**). The percentage of MHCII^+^ CD11c^+^ cells expressing the co-stimulatory molecule CD40 varied between cultures. From days 4-8, two birds consistently contained lower percentage of CD40^+^ cells, regardless of culture conditions (**Figure 3E**) and correlated with the MFI of CD40 expression, with the same two birds having the lowest expression levels (**Figure 3F**). Throughout the culture period, BMMΦ, BMDC and GMDC contained small subpopulations of cells lacking both MHCII and CD11c expression (**Figure S3A**). The MHCII^-^ CD11c^-^ subpopulations were correspondingly low/negative for CD40, MRC1L-B and expressed low levels of the *CSF1R*-tg in all culture conditions (**Figure S3B**). Overall, multi-color flow cytometric analysis indicates that BMDC, GMDC and BMMΦ cultures derived from post-hatch chickens all contained a major cell population expressing MHCII, CD11c and MRC1L-B, but the BMMΦ consistently expressed lower levels of MRC1L-B in comparison to both BMDC and GMDC. Furthermore, addition of IL-4 in BMDC cultures induced no difference in cellular phenotype or expression levels of MPS markers compared to GMDC cultures.

### BMMΦ, BMDC and GMDC have limited phagocytosis abilities

**Figure 4:**
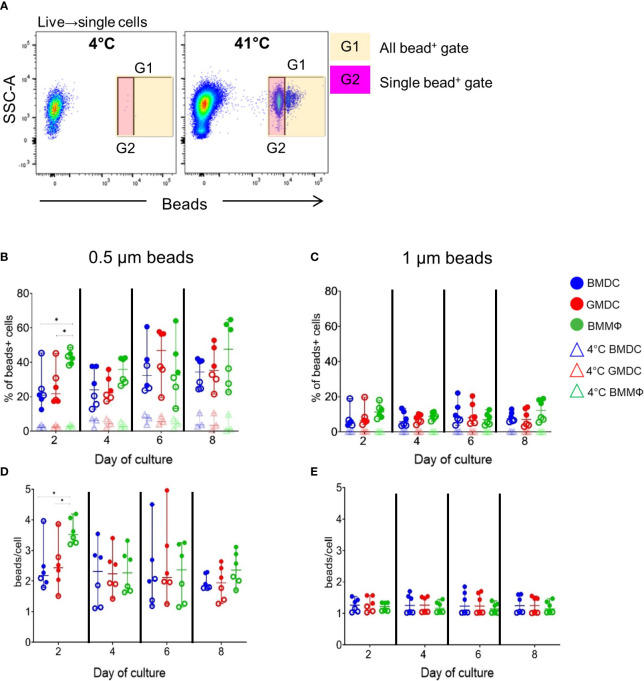
Endocytic activity of BMDC, GMDC and BMMΦ cultures. BMDC (blue), GMDC (red) and BMMΦ (green) were incubated with 0.5 µm or 1 µm fluorescent beads at 41°C (circles) or 4°C (triangles) for 2 h on days 2, 4, 6 and 8 of culture and phagocytosis was analyzed by flow cytometry. **(A)** Representative flow plots of the gating strategy to determine specific bead uptake using the cells treated at 4°C and applied to the cells treated at 41°C. An all bead+ gate (G1) and a gate for cells with a single bead (G2) were applied. **(B)** The percentage of 0.5 µm bead^+^ cells; **(C)** and 1 µm bead^+^ cells; **(D)** the number of 0.5 µm beads per cell; **(E)** the number of 1 µm beads per cell; The number of beads per cell was calculated by dividing MFI of cells in G1 by cells in G2. Data represents the median (± range). From each bird (n=6), BMDC, GMDC and BMMΦ were generated. Data represents two independent experiments (n=3 per experiment, open & closed circles). Significant differences are indicated by *p <0.05.

To determine any functional differences between the cultures, uptake of small (0.5 µm) and large (1 µm) fluorescent beads was analyzed by flow cytometry 2 h post-treatment. To control for non-specific bead adherence, cells were incubated on ice and the percentage of bead^+^ cells and the number of beads per cell were determined (**Figure 4A**). Although only significant at day 2, the BMMΦ had a higher percentage of 0.5 µm bead^+^ cells (**Figure 4B**) compared to the corresponding BMDC and GMDC. Analysis of bead uptake in two independent experiments consisting of three individual chickens, demonstrates large bird-to-bird variation within the cultures (**Figure 4**, filled vs empty circles). In contrast to the percentage of 0.5 µm bead^+^ cells, the percentage of 1 µm bead^+^ cells was much lower across all cell cultures and time points, reaching only a maximum of ~20% and no statistical difference was observed between cultures (**Figure 4C**).

To analyze whether there was a difference across the cultures in the number of beads per cell (beads/cell), the MFI of all bead^+^ cells was divided (**Figure 4A**, gate G1) by the MFI of cells containing a single bead (**Figure 4A**, gate G2). At day 2, BMMΦ contained significantly more 0.5 µm beads/cell (average of 3 beads) compared to the BMDC and GMDC cultures (average of 2 beads). However, from day 4 onwards, the number of 0.5 μm beads/cell did not differ between cultures, and this coincided with the greater variation between two independent experiments. Cultures derived from the same experiment resulted in similar values (percentage bead positive and number beads/cell) suggesting that despite experimental variation there was no functional difference in bead uptake between the three cultures. No significant difference was observed in the number of 1 µm beads/cell between the cultures on each day of analysis and the low percentage of bead+ cells corresponded with a lower number of 1 µm beads/cell (average of 1-2 beads; **Figure 4E**). All BM cultures were more proficient at uptake of 0.5 µm beads compared to 1 µm and BMMΦ were more efficient at uptake on day 2 compared to BMDC and GMDC.

### Phagosomal acidification is highest in BMMΦ cultures on day 2

**Figure 5:**
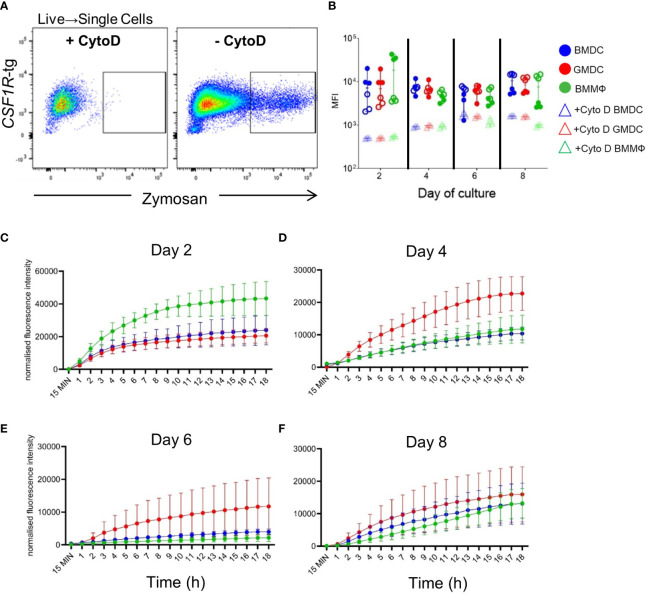
Real-time kinetics of uptake and phagosomal acidification using pHrodo labelled zymosan bioparticles. **(A)** Representative flow plots of the gating strategy. Gates were applied to the cells treated with the actin polymerization inhibitor, Cytochalasin D (CytoD), as negative controls. **(B)** MFI of zymosan in CytoD treated (rectangle) or untreated (circles) BMDC (blue), GMDC (red) and BMMΦ (green) on days 2, 4, 6 and 8 of culture by flow cytometry. Data represents the median (± range). From each bird (n=6), BMDC, GMDC and BMMΦ were generated. Data represents two independent experiments (n=3 per experiment, open & closed circles). **(C–F)** Real time kinetics of BMDC, GMDC and BMMΦ phagosomal acidification 15 min, and at every hour up to 18 h post-treatment with zymosan bioparticles. Data are represented as mean ( ± SD) of three biological replicates normalized to background fluorescence of cells treated with CytoD.

Cell cultures were analyzed for their ability to uptake and acidify pH sensitive pHrodo labelled zymosan bioparticles. To control for non-specific zymosan uptake, cells were treated with an actin-polymerization inhibitor, CytoD, for 30 min prior to a 2 h incubation with zymosan (**Figure 5A**). All cells were capable of acidifying zymosan bioparticles from day 2 of culture and there was no significant differences in the MFI across the days or between the cultures (**Figure 5B**).

To determine if the kinetics of phagosomal acidification may be associated with functional differences between the cell cultures, the levels and increase in fluorescence were measured every hour for 18 h on days 2, 4, 6 and 8 (**Figures 5C–F**). On day 2 of culture, there was a rapid increase in fluorescence in all cultures, with the fluorescent intensity in BMMΦ higher than in BMDC and GMDC (**Figure 5C**). On days 4 and 6, a higher increase in acidification was observed in the GMDC compared to the BMDC and BMMΦ (**Figures 5D, E**). On days 6 and 8 of culture, the fluorescence intensities were much lower compared to day 2 (**Figures 5E, F**), while in day eight cultures there was no differences observed between the cultures. Overall, no difference in the level and speed of acidification was observed across the cultures.

### NO production decreases over time in BMMΦ

**Figure 6:**
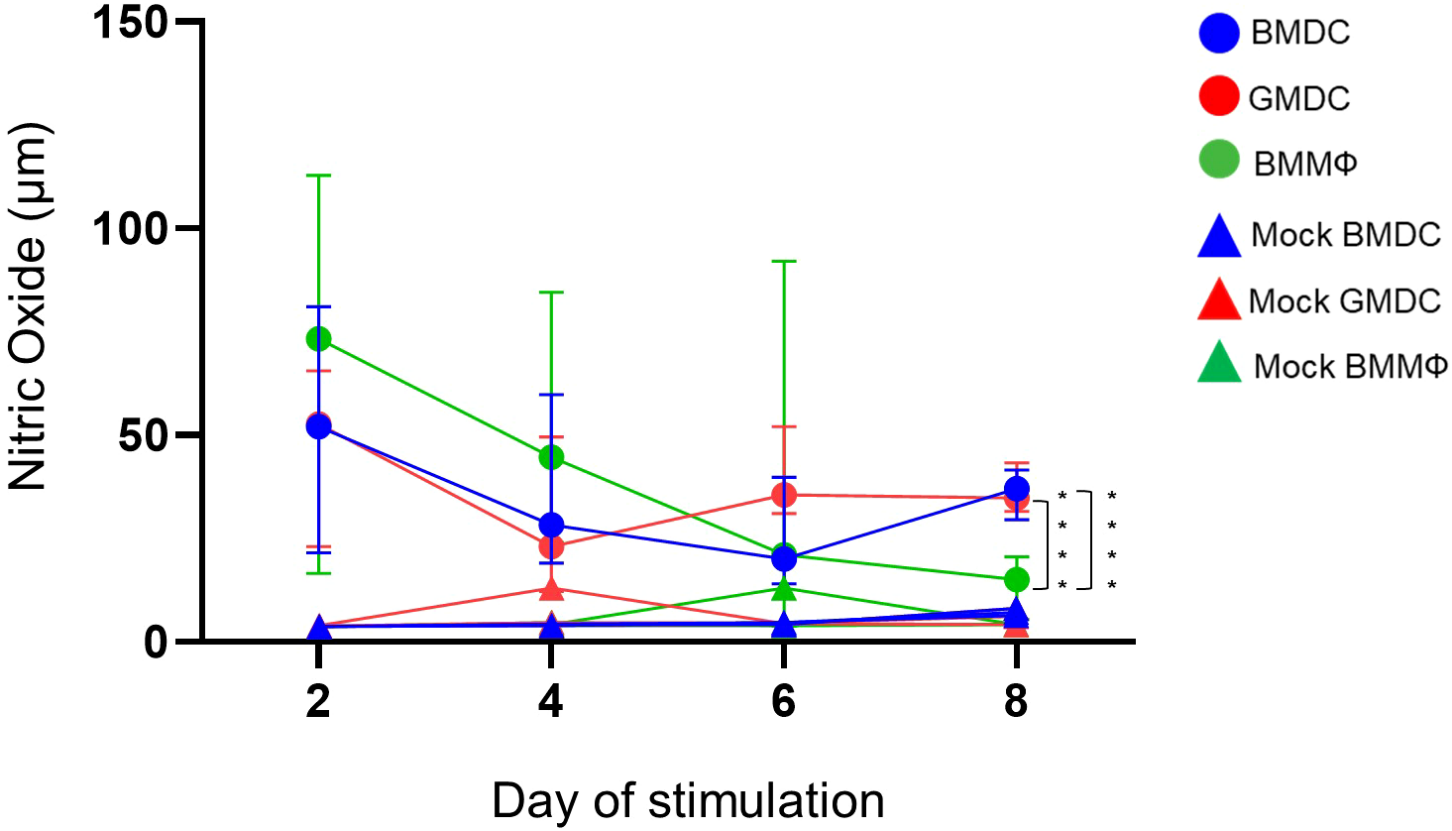
BMDC and GMDC produce high NO levels post-LPS treatment. BM cultures were stimulated on days 2, 4, 6 and 8 with LPS from *E*. *coli* (circles) or untreated (Mock, rectangles). NO levels in cell supernatant were analyzed 48 h post-treatment using Griess reaction. Data represents the median (± range). From each bird (n=6), BMDC, GMDC and BMMΦ were generated. Data represents two independent experiments (n=3 per experiment, open & closed circles). Significant differences between the cultures on the day of stimulation are indicated by * p<0.05 and ****p<0.001.

NO production by the BMDC, GMDC and BMMΦ was analyzed after LPS stimulation on days 2, 4, 6 and 8 of culture. Forty-eight hours post-LPS stimulation all cell cultures produced NO compared to unstimulated cells (**Figure 6**), but no significant difference in NO production between the cell cultures was observed on day 2 and 4. However, on day 6, the GMDC produced significantly higher levels of NO compared to BMDC. There was significant variation in NO production by BMMΦ and the variation observed within the cultures did not correlate with any specific individual bird/culture i.e high responders on particular time point were not correlated with high responders at the following time point. At day 8, NO production was highly consistent across the individual birds within each cell culture and both the BMDC and GMDC produced significantly higher levels of NO compared to BMMΦ (**Figure 6**).

### Temporal transcriptomic analysis suggest BMDC, GMDC and BMMΦ differentiate to the macrophage cell lineage

**Figure 7:**
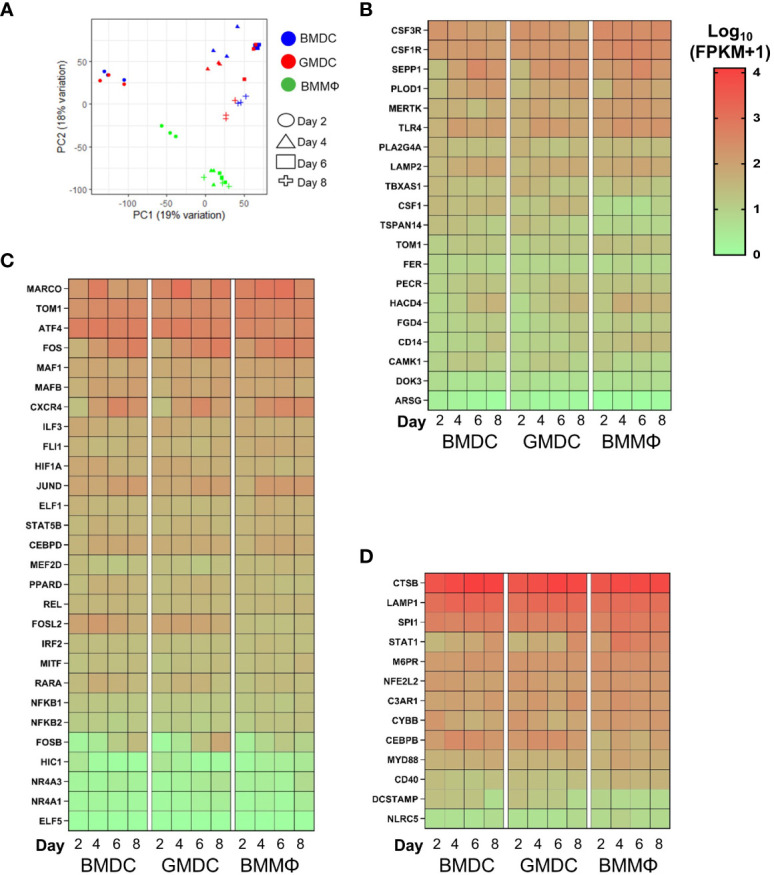
Chicken bone marrow cultures are enriched for monocyte/macrophages related gene signatures. **(A)** PCA of global gene expression profiles of BM cultures on days 2, 4, 6 and 8 of culture. Heat maps of gene expression levels of **(B)** core macrophage genes; **(C)** monocyte/macrophages related genes; **(D)** myeloid effector/receptor genes and lineage related transcription factors in BMDC, GMDC and BMMΦ at days 2, 4, 6 and 8 of culture. Heat maps are represented as the median of Log10 transformed Fragments Per Kilobase of transcript per Million mapped reads (FPKM) values from three independent biological replicates on each day from each culture. High reads shown in red and low reads in green.

To define the enriched MPS cell lineage and their dynamics in the chicken bone marrow cultures grown under the influence of different cytokines, RNA–seq analysis was performed on the day 2, 4, 6 and 8 cultures. Samples for RNA-seq were preferential towards the adherent and semi-adherent cells in each culture as floating cells were discarded during sample preparation. Firstly, PCA identified six clusters, grouping day 2 BMDC and GMDC together and separated from day 2 BMMΦ (**Figure 7A**). Day 4 to 8 BMMΦ clustered together whereas the BMDC and GMDC cultures were clustered based on the specific culture day.

Next, each culture was analyzed over time for the enrichment of monocyte/macrophage and DC cell lineage genes, related transcription factors, myeloid receptors and lysosomal components previously identified in mammals (19, 31, 32) and in chicken (21, 22, 33, 34) (**Figures 7B–D**). Firstly, the enrichment of core macrophage related genes showed little differences in expression between the cell cultures (**Figure 7B**). *CSF3R*, *MERTK*, *SEPP1* and *TLR4* were expressed at modestly higher levels in the BMMΦ compared to the BMDC and GMDC cultures. *CD14*, *FGD4*, *HACD4*, *PECR* and *TOM1* were expressed in a temporal fashion, with expression levels increasing with time in BMDC and GMDC cultures (**Figure 7B**). The enrichment of monocyte/macrophage related genes further demonstrated no major differences in expression levels across the cultures (**Figure 7C**). The most notable differences were observed for FOS gene family, with *FOS* mRNA expression levels increasing with time in BMDC and GMDC in comparison to BMMΦ cultures. *FOSL2* was expressed at higher levels in the DC cultures compared to the BMMΦ cultures irrespective of time point, an expression trend also observed for *RARA. FOSB* mRNA expression levels were also higher in the BMDC and GMDC cultures compared to BMMΦ on days 6 and 8 (**Figure 7C**). The macrophage-associated transcription factors *SPI1* and *STAT1* were expressed slightly higher in BMMΦ, contrastingly *CEBPB* expression levels were elevated in BMDC and GMDC (**Figure 7D**).To further distinguish transcriptomic differences between the BM cultures, a number of genes related to myeloid effector and receptor functions were analyzed. Similarly, little differences in expression levels of these genes were observed between and over time in the cultures (**Figure 7D**).

**Figure 8:**
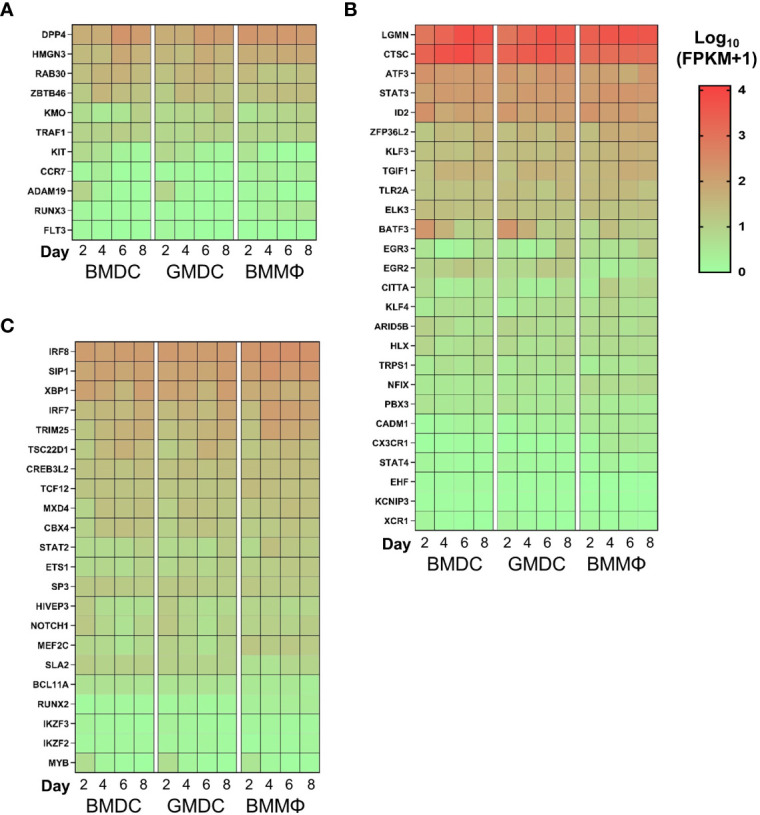
Chicken BM cultures are not enriched for DC related transcripts. Heat maps of gene expression levels of **(A)** pDC; **(B)** cDC and **(C)** core DC markers. Heat maps are represented as the median of Log10 transformed Fragments Per Kilobase of transcript per Million mapped reads (FPKM) values from three independent biological replicates on each day from each culture. High reads shown in red and low reads in green.

We next analyzed the expression levels of DC related genes across the cultures over time. Interestingly, no enrichment of a core set of DC related genes were observed in any of the cultures over time (**Figure 8A**). Analysis of cDC related genes demonstrated the high level of expression of *CTSC* and *LGMN* across all cultures. However, there was little difference in the expression of key cDC related transcription factors such as *ID2* or *IRF8*, cell markers such as *CADM1* and *XCR1 *and the majority of cDC related genes were expressed at low levels in all cultures over time. Of note, *BAFT3* expression decreased over time in the BMDC and GMDC cultures (**Figure 8B**). In addition, pDC related gene signatures were not enriched in any of the cultures over time (**Figure 8C**). Overall, chicken bone marrow cells grown under lineage determining cytokines are biased towards macrophage lineage development with no DC gene signature enrichment observed in any of the cultures.

### BMDC and GMDC developmental pathway towards macrophages is slower compared to BMMΦ

**Figure 9:**
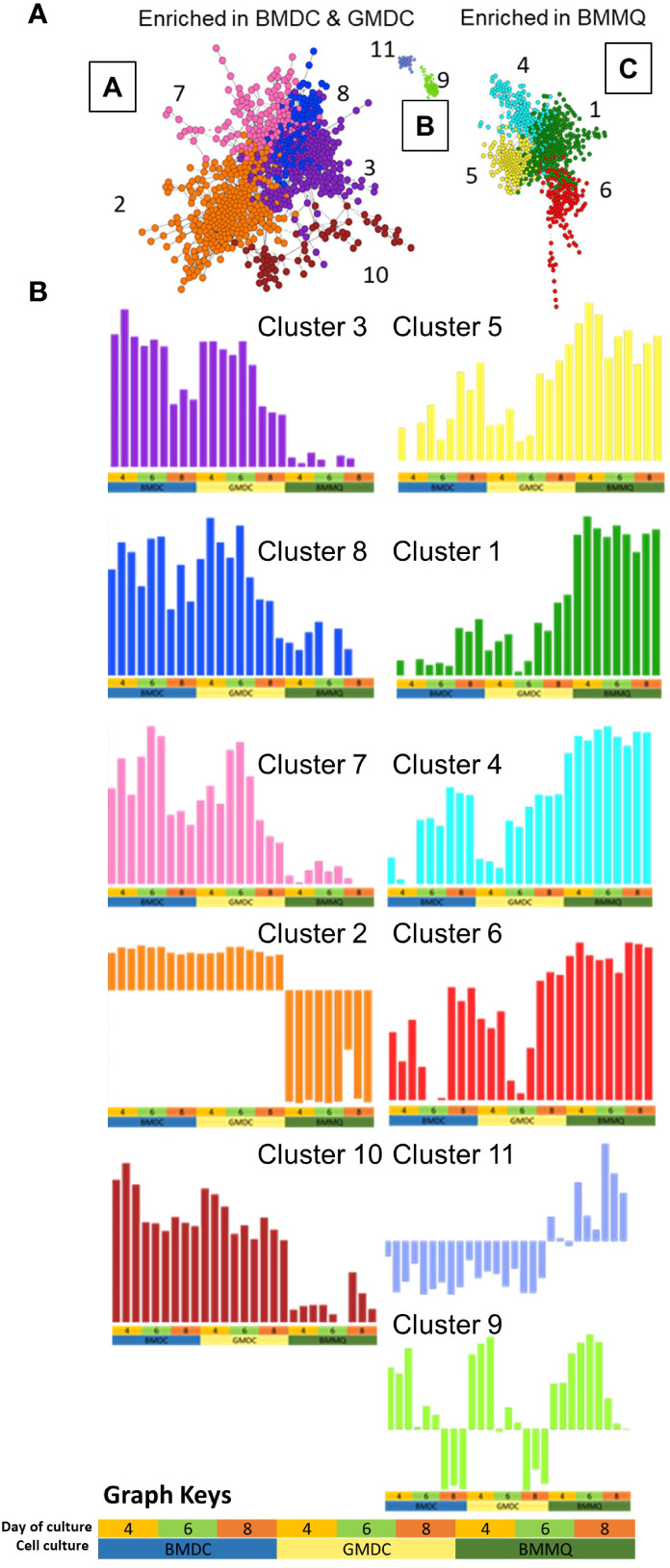
Gene-gene network graphs. **(A)** Sample-sample network graph. The graph is colored based on experimental groups and shows 3 clusters **(A–C)** comprised of 11 separate clusters (1-11). Each sphere (node) represents a gene, and lines between them (edges) show Pearson correlations between them of ≥0.95. Primary separation of samples is based on culture type. **(B)** Gene-gene network graphs demonstrating the expression pattern of the genes within the 11 clusters (1-11). The culture type and day of culture are denoted by the key at the base of the figure and used under each graph.

In order to compare global gene expression profiles, relationships between the cultures over time and examination of differences observed in the PCA, pairwise Pearson correlations (r) were calculated between all the transcripts using Graphia (**Figure 9**). The day 2 cultures sample-to-sample analysis revealed a time effect relative to all other samples, and therefore the data were excluded in further analysis. A network graph was constructed using a correlation threshold r=0.95 producing three clusters (A-C). Cluster A was enriched for the BMDC and GMDC cultures while cluster C was enriched for the BMMΦ cultures. Using Louvain clustering algorithm co-expression clusters were compiled and analyzed using gene ontology databases. The enrichment profiles of each cluster are provided in **File S1**. Genes in clusters 3, 7, 8 and 10 were highly expressed in BMDC and GMDC whereas they were lowly expressed in BMMΦ. These clusters consisted of genes involved in protein modification processes (Cluster 3; *AK9, DHTKD1, ENO2, INPP5K*, *MRPL13, PKM2, PGMA1*), cellular metabolic processes, such as glucose metabolism (*HK1 HK2, H6PD, GPI, LDHA*), amino acid metabolism (Cluster 10; *ASNS, GPD2, SARS, SCL7A1, SCL7A11, YARS*) and repressor of *KLF4* expression (*ZNF706*). Cluster 7 also consisted of genes involved in the positive regulation of leukocyte activation (*CD274, ENO1, HES1, KARS, PRELID1*) and autophagy (*ARMC8, ATG4B, EIF3E, EXOG, TPI1, Ufd1l*). Genes within cluster 2 were expressed at low levels in BMDC and GMDC and even further downregulated in BMMΦ. This cluster consisted of genes involved in the regulation of cell adhesion (*CLDN5*, *HRSA*, *ITGA6, LGAL53*, *VEGFA*, *VEGFC*).

Genes within clusters 1, 4, 5 and 6 were expressed at higher levels in the BMMΦ with little difference in expression levels across the time-points analyzed (**Figure 9B**). In contrast, the genes within these clusters were expressed in a temporal fashion, increasing with time in BMDC and GMDC and consisted of genes related to leukocyte activation and immune system processes (Cluster 1; *B2M, CARD11*, *CCR5, CD40*, *FLI1, IKZF1, IRF7, IRF8, LY86, NFATC2*, *NFKB2*, *NKBKB2, PLCG2,TAP1, TAP2, TGFB1, TNFSF10)*, anti-viral immune responses (Cluster 5; *IFI35, IFIH1, EIF2AK2, MyD88, STAT1, STAT2, TRIM25*). The clusters also consisted of genes involved in the complement cascade (*C1QA, C1QB, C1QC*), lysosomal function (Cluster 4; *ACP5, ASAH1, CTSA, CTSH, CYB561A3, GLA, NEU3, SNX14*, *VPS11*) and mRNA stability (Cluster 6; *DHX36, HNRNPM, HNRNPR, TAF15, ZC3H14*).

Cluster 11 represented a profile that was downregulated in BMDC and GMDC and increased over time in BMMΦ. It comprised of genes involved in endothelial cell proliferation (*BMPER, LOXL2*) and regulation of endodermal cell differentiation (*COL5A1/6A/6A1/6A3/12A1*). Genes within cluster 9 had a pronounced time-dependent co-expression relationship, decreasing overtime in BMDC and GMDC while increasing from day 4-6 in BMMΦ and decreasing by day 8 (**Figure 9**). Genes within this cluster are related to the cell cycle and DNA replication processes (*CENPF*/*I*/*K*/*L*/*N*/*P*/*W*, *KIF11*/*14*/*18A*/*20A23*/*2C* and *MCM5/6/10*). Network gene analysis demonstrated key differences in the temporal expression of genes involved in cellular functions between BMMΦ and BMDC, GMDC cultures. It further demonstrated that the addition of IL-4 to BMDC induced no significant global transcriptional differentiation from GMDC.

## Discussion

Chicken MPS cells derived from the BM permit investigation of key cellular functions including scavenging of dying cells, pathogens, and molecules through phagocytosis, endocytosis and T cell activation, making these models vital for understanding their contribution to immunity and inflammatory responses to pathogenic microorganisms. To obtain large numbers of macrophages or cDC, BM progenitor cells are cultured in the presence of hematopoietic cytokines CSF1, CSF2 + IL-4, to drive macrophage or DC development, respectively (26, 28, 35–37). Recent studies have shown that the addition of FLT3L to BM cultures can promote the growth of cDC (11, 16). However, biologically active chicken FLT3L is currently unavailable and therefore its role in cDC development in chicken BM cells was not analyzed in this study. To this end, a thorough investigation of chicken BM cultures treated with the traditional CSF2 + IL-4 or with CSF2 or CSF1 alone, was carried out to understand the heterogeneity across the cultures, their function and cell lineage investigated by temporal transcriptomic analysis.

The phenotypical characterization of mammalian DC and macrophages and their distinction from each other are associated with expression of several marker genes. Higher expression of MHCII and CD11c are historically associated with the DC phenotype, related to their antigen-presenting abilities (6). However, such phenotypical differences were not present in our chicken BM cultures, irrespective of cytokines used. In a recent study, GMDC-derived from ED18 chick embryos, consisted of MHCII^low^ and MHCII^high^ subpopulations (36). This study found that both the species origin and concentration of serum used can alter the phenotype of the BM cells grown with CSF2, as the MHCII^low^ subpopulation was absent in the 10% FBS treated cultures compared to the 5% chicken serum treated cultures (36). In our study, 10% FBS was utilized, potentially lacking adult chicken-derived serum components, that may drive development of the MHCII^low^ subpopulation. Phenotypical analysis found the percentage of MHCII^+^ CD11c^+^ subpopulation was significantly higher in BMMΦ on day 2 compared to BMDC and GMDC. Interestingly, the mannose receptor, MRC1L-B, expression levels were significantly lower on the BMMΦ at each day of analysis compared to both BMDC and GMDC. In the chicken spleen, MRC1L-B is highly expressed by macrophages and either lowly expressed or lacking in cDC (21, 23, 25). However, murine and human BM or monocyte derived-DC express the mannose receptor (MR, also known as CD206) (38). It has been reported that IL-4 increases MR expression on peritoneal macrophages, which is normally down-regulated by IFN-γ treatment (39). Our previous study showed a reduction in MRCL1-B expression in chicken BMMΦ post-LPS treatment, hence its level of expression may indicate maturation status in chicken BMMΦ (40). Murine BM cultured with CSF2 can include macrophages, in addition to DC and neutrophils (2). A small population of MHCII^-^ CD11c^-^ *CSF1R*-tg^lo^ cells was evident in each culture indicating the presence of granulocytes in the chicken BM cell cultures (30). We also detected *CSF1R*-tg^-^ MRCL1-B^+^ cells in all cultures, a subpopulation previously observed in the chicken spleen (23) and more recently in BMDC (25).

Both phagocytic and acidification assays across the cultures indicated no differences in the ability of the cells to uptake and undertake luminal acidification of phagosomes. Low levels of 1 µm bead uptake by the BM cultures in our study coincides with previously observed receptor-mediated uptake of IgY-coated 1 µm beads in GMDC (36). Similar levels of zymosan bioparticle uptake and acidification was observed across all cultures. It may be plausible that for more efficient phagocytosis of larger particles TLR activation in chicken BM cultures is required, similarly to mammals (41). Targeting C-type lectins, such as dectin-1, has been showed to increase the phagocytosis capacity of BMDC (37). Recently, exposure of chicken splenic cDCs (FLT3^Hi^) and macrophages (FLT3^low-neg^) to non-invasive *Salmonella* Typhimurium also resulted in equally efficient phagocytosis by both cell types (25), suggesting that both cell populations have the capacity to uptake larger particles. The response of cultures to LPS, measured *via* NO production, shown no significant difference between the cultures and levels decrease with time. This coincides with a previous study in which little difference in pro-inflammatory cytokine mRNA expression levels was observed between LPS treated BMMΦ and BMDC (40). Although attempts were made to compare the antigen-presenting abilities of the BMDC, GMDC and BMMΦ using syngenic and allogenic T cells, T cell proliferation was only achieved after stimulation with ConA (data not shown). Reproducible T cell proliferation assays in the chicken using antigen-presenting cells still requires further development.

Comparative transcriptomic analysis of immune cells between mammals and chickens provides a framework to support model systems in the context of MPS biology (1, 18, 20, 42). Chicken blood monocytes, tissue-resident macrophages and cDC have been described based on their expression of conserved mammalian cell lineage related genes (21–24). RNA-seq analysis of CSF1-treated chicken BM cells has demonstrated their macrophage lineage by day 7 of culture (22, 30). The transcriptome of CSF1-treated chicken BM cells were further analyzed in this study on days 2, 4, 6 and 8 and used to examine the enriched cell lineages along with BM treated with CSF2 + IL-4 or CSF2 alone. Firstly, the BM cell culture transcriptome were analyzed for core macrophage and DC related gene signatures on days 2, 4, 6 and 8 of culture. Interestingly, a core macrophage-lineage related gene signature (*CEBPB, CSF1R, NFE2L2, MAFB*, *STAT1, TLR4*) (22, 31, 43–45) along with lysosomal components (*CTSB*, *LAMP1/2*, *M6PR*) (46, 47) were found to be highly expressed across all culture conditions, a co-expression that suggests a core macrophage transcriptional program is being driven in all cultures. In addition, sustained levels of *SPI1* (*Pu.1*) expression was observed across all time-points and cultures, a transcription factor that is required at high levels to induce and maintain macrophage differentiation (48). When analyzing cDC related gene enrichment in all cultures, (*FLT3*, *BATF3, CADM1*, *XCR1*, *Zbtb46*) (49–54), the highest expressions levels were observed in BMDC and GMDC on day 2. This may indicate that without FLT3L, chicken BM-derived DC do not differentiate or have longevity in culture. Since this study, Wu et al. (25) confirmed the absence of FLT3 and XCR1 protein expression on day 7 BMDC. FOS genes (*FOS, FOSB, FOSL2*) are involved in osteoclast formation and their high expression indicates the presence of these cell lineages in the cultures (55, 56). Colony-stimulating factors are known to “prime” or “activate” macrophages as well as induce their differentiation (57). Our data further demonstrates that BM cells treated with CSF2 + IL-4 or CSF2 alone induce a core macrophage transcriptional program similar to CSF1 treated cells.

Network gene analysis demonstrated key differences in the temporal expression of genes with related functions across the cultures. At all time-points analyzed, BMMΦ expressed high levels of MHCI related genes (*B2M, TAP1, TAP2*) (22), genes involved in immune regulation, such as transcriptional control (*IFI35, IKZF1, IRF7, IRF8, FLI, NFATC2*, *NFKB2*, *NKBKB2, STAT2*) and immune function (*CARD11, CCR5, CD40*, *EIF2AK2, IFIH1, IL1R2, IL31RA, LY86, MyD88, PLCG2, TGFB1, TNFAIP8L2, TNFSF10*, *TRIM25*) (58). In contrast, the expression levels of these genes increased with time in BMDC and GMDC cultures, with the highest levels observed at day 8, demonstrating their developmental progress towards a similar transcriptome as BMMΦ.

Macrophage metabolic requirements change with development and functional polarization (59). Genes involved in cellular metabolic processes, including glucose transport (*HK1* and *HK2*), amino acid metabolism and solute transport, (*ENO1, SCL7A1, SCL7A11, SLC16A3, SLC16A4, SLC25A17*) (60, 61) were expressed at higher levels in BMDC and GMDC compared to BMMΦ throughout the developmental period analyzed. This may indicate that the BMDC and GMDC are more metabolically active compared with BMMΦ, potentially linked to their ongoing differentiation to a macrophage, or reflect the heterogeneity of cells in the culture (59). Macrophages are sub-categorized into either pro-inflammatory M1 macrophages, polarized by LPS or IFN-γ, or anti-inflammatory M2 macrophages, polarized by IL-4 or IL-13 (62–64). KLF4 can differentially affect a repertoire of genes that characterize the M1 and M2 phenotype. With respect to M2 polarization, following IL-4 stimulation, KLF4 and STAT6 synergistically induce M2 gene targets, such as Arg-1 (65). The high expression of *ZNF706*, an inhibitor of KLF4 transcription, in BMDC and GMDC, indicates that these cultures may not represent M2 macrophages. Interestingly, we observed an enrichment of collagen related genes (COL5A1/6A/6A1/6A3/12A1) and low expression of cell-adhesion related genes (*CLDN5*, *HRSA*, *ITGA6, LGAL53*, *VEGFA*, *VEGFC*) in all cultures. While macrophages can produce proteases to degrade and destabilize the extracellular matrix, they can also produce collagen to maintain tissue integrity, which may encourage adherence along with macrophage-leukocyte communication (66). It should be noted that BM-derived culture protocols not only differ in the concentration of differentiation factors and duration of growth, but also the inclusion of loosely and non-adherent cells. In rodents BM cultures, non-adherent cells consisted of mature DC, whereas adherent cells consisted of tolerogenic DC and firmly adherent cells were considered macrophages (67). In our study, the adherent and loosely attached cells were included in characterization of the cultures. Therefore, future studies should consider whether the morphological heterogeneous nature of the BMDC and GMDC cultures could account for different cell lineages. Overall, RNA-seq transcriptomic and network gene analysis indicates that chicken BM cultures treated with CSF1, CSF2 + IL-4 or CSF2 alone activate a core macrophage transcriptional program. Whilst BMMΦ have a more “primed” macrophage within 2 days of culture, the developmental pathway towards a macrophage is somewhat delayed in BMDC and GMDC. In addition, subtle differences between the transcriptomes of BMDC and GMDC suggests they follow a similar development pathway irrespective of the presence of IL-4.

FLT3L-derived porcine BM-derived cell cultures supported the development of three distinct cell populations based on expression of CADM1, CD14, MHCII and CD172a defining putative cDC1, cDC2 and a novel CD14^+^ cell population (11). FLT3L treated bone marrow cultures in chicken have not been developed, but may lead to the *in vitro* generation of bona fide chicken cDC. Simplified *in vitro* BM-derived cell models can be meaningful; however, these cell sources to study tissue-derived MPS biology should be met with caution as tissue-specific factors alter the function of MPS cells in mammals (32). With the advent of unbiased single cell sequencing technology, the ability to identify more heterogeneity within the MPS in healthy and diseased animals is now becoming more achievable.

## Data availability statement

The datasets generated and analyzed for this study are included in the published article (and its additional files) or in the following data repository. The RNA-seq data for this study was deposited in the European Nucleotide Archive (ENA) at EMBL-EBI under accession number PRJEB56177.

## Ethics statement

The animal study was reviewed and approved by The Roslin Institute, University of Edinburgh, Ethical Review Committee.

## Author contributions

DB, KS, and LV conceptualized the study. DB, KS, and LV performed or assisted with the experiments. DB performed the data analysis. SS contributed to the transcriptome analysis. DB, KS and LV wrote the manuscript. LV secured the funding. All authors contributed to, read and approved the final manuscript.

## References

[1] T'Jonck W Guilliams M Bonnardel J. Niche signals and transcription factors involved in tissue-resident macrophage development. Cell Immunol (2018) 330:43–53. doi: 10.1016/j.cellimm.2018.02.005 29463401 PMC6108424

[2] Inaba K Inaba M Romani N Aya H Deguchi M Ikehara S. Generation of Large numbers of dendritic cells from mouse bone marrow cultures supplemented with Granulocyte/Macrophage colony-stimulating factor. J Exp Med (1992) 176(6):1693–702. doi: 10.1084/jem.176.6.1693 PMC2119469 1460426

[3] Swiecki M Gilfillan S Vermi W Wang Y Colonna M. Plasmacytoid dendritic cell ablation impacts early interferon responses and antiviral nk and Cd8(+) T cell accrual. Immunity (2010) 33(6):955–66. doi: 10.1016/j.immuni.2010.11.020 PMC3588567 21130004

[4] Stanley ER Chen DM Lin HS. Induction of macrophage production and proliferation by a purified colony stimulating factor. Nature (1978) 274(5667):168–70. doi: 10.1038/274168a0 307187

[5] Lardon F Snoeck HW Berneman ZN Van Tendeloo VF Nijs G Lenjou M. Generation of dendritic cells from bone marrow progenitors using gm-csf, tnf-alpha, and additional cytokines: Antagonistic effects of il-4 and ifn-gamma and selective involvement of tnf-alpha receptor-1. Immunology (1997) 91(4):553–9. doi: 10.1046/j.1365-2567.1997.00295.x PMC1363875 9378494

[6] Helft J Bottcher J Chakravarty P Zelenay S Huotari J Schraml BU. Gm-csf mouse bone marrow cultures comprise a heterogeneous population of Cd11c(+)Mhcii(+) macrophages and dendritic cells. Immunity (2015) 42(6):1197–211. doi: 10.1016/j.immuni.2015.05.018 26084029

[7] Jung S Unutmaz D Wong P Sano G De los Santos K Sparwasser T. *In vivo* depletion of Cd11c+ dendritic cells abrogates priming of Cd8+ T cells by exogenous cell-associated antigens. Immunity (2002) 17(2):211–20. doi: 10.1016/s1074-7613(02)00365-5 PMC3689299 12196292

[8] Enders M Franken L Philipp M-S Kessler N Baumgart A-K Eichler M. Splenic red pulp macrophages cross-prime early effector ctl that provide rapid defense against viral infections. J Immunol (2020) 204(1):87–100. doi: 10.4049/jimmunol.1900021 31776205

[9] Schliehe C Redaelli C Engelhardt S Fehlings M Mueller M van Rooijen N. CD8– dendritic cells and macrophages cross-present poly (D, l-Lactate-Co-Glycolate) acid microsphere-encapsulated antigen in vivo. J Immunol (2011) 187(5):2112–21. doi: 10.4049/jimmunol.1002084 21795597

[10] Daniel SL Legendre AM Moore RN Rouse BT. Isolation and functional studies on feline bone marrow derived macrophages. Vet Immunol Immunopathol (1993) 36(2):107–22. doi: 10.1016/0165-2427(93)90101-9 PMC7119569 8475618

[11] Li Y Puebla-Clark L Hernández J Díaz I Mateu E. Development of pig conventional dendritic cells from bone marrow hematopoietic cells in vitro. Front Immunol (2020) 11:553859. doi: 10.3389/fimmu.2020.553859 33162975 PMC7580533

[12] Tipold A Zurbriggen A Moore P Schijns V Jungi TW. Generation and functional characterisation of canine bone marrow-derived macrophages. Res Vet Sci (1998) 64(2):125–32. doi: 10.1016/s0034-5288(98)90007-8 9625468

[13] Foulon E Foucras G. Two populations of ovine bone marrow-derived dendritic cells can be generated with recombinant gm-csf and separated on Cd11b expression. J Immunol Methods (2008) 339(1):1–10. doi: 10.1016/j.jim.2008.07.012 18718839

[14] Lutz MB Kukutsch N Ogilvie AL Rossner S Koch F Romani N. An advanced culture method for generating Large quantities of highly pure dendritic cells from mouse bone marrow. J Immunol Methods (1999) 223(1):77–92. doi: 10.1016/s0022-1759(98)00204-x 10037236

[15] Na YR Jung D Gu GJ Seok SH. Gm-csf grown bone marrow derived cells are composed of phenotypically different dendritic cells and macrophages. Mols Cells (2016) 39(10):734–41. doi: 10.14348/molcells.2016.0160 PMC5104881 27788572

[16] Brasel K De Smedt T Smith JL Maliszewski CR. Generation of murine dendritic cells from Flt3-Ligand-Supplemented bone marrow cultures. Blood (2000) 96(9):3029–39. doi: 10.1182/blood.V96.9.3029 11049981

[17] Guzylack-Piriou L Alves MP McCullough KC Summerfield A. Porcine Flt3 ligand and its receptor: Generation of dendritic cells and identification of a new marker for porcine dendritic cells. Dev Comp Immunol (2010) 34(4):455–64. doi: 10.1016/j.dci.2009.12.006 20015454

[18] Guilliams M Ginhoux F Jakubzick C Naik SH Onai N Schraml BU. Dendritic cells, monocytes and macrophages: A unified nomenclature based on ontogeny. Nat Rev Immunol (2014) 14(8):571–8. doi: 10.1038/nri3712 PMC4638219 25033907

[19] Gautier EL Shay T Miller J Greter M Jakubzick C Ivanov S. Gene-expression profiles and transcriptional regulatory pathways that underlie the identity and diversity of mouse tissue macrophages. Nat Immunol (2012) 13(11):1118–28. doi: 10.1038/ni.2419 PMC3558276 23023392

[20] Guilliams M Dutertre C-A Scott CL McGovern N Sichien D Chakarov S. Unsupervised high-dimensional analysis aligns dendritic cells across tissues and species. Immunity (2016) 45(3):669–84. doi: 10.1016/j.immuni.2016.08.015 PMC5040826 27637149

[21] Vu Manh TP Marty H Sibille P Le Vern Y Kaspers B Dalod M. Existence of conventional dendritic cells in gallus gallus revealed by comparative gene expression profiling. J Immunol (2014) 192(10):4510–7. doi: 10.4049/jimmunol.1303405 24740508

[22] Hu T Wu Z Bush SJ Freem L Vervelde L Summers KM. Characterization of subpopulations of chicken mononuclear phagocytes that express Tim4 and Csf1r. J Immunol (2019) 202(4):1186–99. doi: 10.4049/jimmunol.1800504 PMC6436730 30626692

[23] Sutton KM Morris KM Borowska D Sang H Kaiser P Balic A. Characterization of conventional dendritic cells and macrophages in the spleen using the Csf1r-reporter transgenic chickens. Front Immunol (2021) 12:636436. doi: 10.3389/fimmu.2021.636436

[24] Alber A Morris KM Bryson KJ Sutton KM Monson MS Chintoan-Uta C. Avian pathogenic escherichia coli (Apec) strain-dependent immunomodulation of respiratory granulocytes and mononuclear phagocytes in Csf1r-reporter transgenic chickens. Front Immunol (2019) 10:3055. doi: 10.3389/fimmu.2019.03055 31998322 PMC6967599

[25] Wu Z Hu T Chintoan-Uta C Macdonald J Stevens MP Sang H. Development of novel reagents to chicken Flt3, Xcr1 and Csf2r for the identification and characterization of avian conventional dendritic cells. Immunology (2022) 165(2):171–94. doi: 10.1111/imm.13426 PMC10357484 34767637

[26] Garceau V Balic A Garcia-Morales C Sauter KA McGrew MJ Smith J. The development and maintenance of the mononuclear phagocyte system of the chick is controlled by signals from the macrophage colony-stimulating factor receptor. BMC Biol (2015) 13:12. doi: 10.1186/s12915-015-0121-9 25857347 PMC4369834

[27] Garceau V Smith J Paton IR Davey M Fares MA Sester DP. Pivotal advance: Avian colony-stimulating factor 1 (Csf-1), interleukin-34 (Il-34), and csf-1 receptor genes and gene products. J Leukoc Biol (2010) 87(5):753–64. doi: 10.1189/jlb.0909624 20051473

[28] Wu Z Rothwell L Young JR Kaufman J Butter C Kaiser P. Generation and characterization of chicken bone marrow-derived dendritic cells. Immunology (2010) 129(1):133–45. doi: 10.1111/j.1365-2567.2009.03129.x PMC2807494 19909375

[29] Freeman TC Horsewell S Patir A Harling-Lee J Regan T Shih BB. Graphia: A platform for the graph-based visualisation and analysis of high dimensional data. PloS Comput Biol (2022) 18(7):e1010310. doi: 10.1371/journal.pcbi.1010310 35877685 PMC9352203

[30] Balic A Garcia-Morales C Vervelde L Gilhooley H Sherman A Garceau V. Visualisation of chicken macrophages using transgenic reporter genes: Insights into the development of the avian macrophage lineage. Development (2014) 141(16):3255–65. doi: 10.1242/dev.105593 PMC4197536 25063453

[31] Bakri Y Sarrazin S Mayer UP Tillmanns S Nerlov C Boned A. Balance of mafb and pu. 1 specifies alternative macrophage or dendritic cell fate. Blood (2005) 105(7):2707–16. doi: 10.1182/blood-2004-04-1448 15598817

[32] Mahiddine K Hassel C Murat C Girard M Guerder S. Tissue-specific factors differentially regulate the expression of antigen-processing enzymes during dendritic cell ontogeny. Front Immunol (2020) 11:453. doi: 10.3389/fimmu.2020.00453 32296417 PMC7136460

[33] Vu Manh TP Elhmouzi-Younes J Urien C Ruscanu S Jouneau L Bourge M. Defining mononuclear phagocyte subset homology across several distant warm-blooded vertebrates through comparative transcriptomics. Front Immunol (2015) 6:299. doi: 10.3389/fimmu.2015.00299 26150816 PMC4473062

[34] Bush SJ McCulloch MEB Lisowski ZM Muriuki C Clark EL Young R. Species-specificity of transcriptional regulation and the response to lipopolysaccharide in mammalian macrophages. Front Cell Dev Biol (2020) 8:661. doi: 10.3389/fcell.2020.00661 32793601 PMC7386301

[35] de Geus ED Tefsen B van Haarlem DA van Eden W van Die I Vervelde L. Glycans from avian influenza virus are recognized by chicken dendritic cells and are targets for the humoral immune response in chicken. Mol Immunol (2013) 56(4):452–62. doi: 10.1016/j.molimm.2013.06.007 23911401

[36] van den Biggelaar RHGA Arkesteijn GJA Rutten VPMG van Eden W Jansen CA. *In vitro* chicken bone marrow-derived dendritic cells comprise subsets at different states of maturation. Front Immunol (2020) 11. 141:141. doi: 10.3389/fimmu.2020.00141 32174908 PMC7054383

[37] Larsen FT Guldbrandtsen B Christensen D Pitcovski J Kjaerup RB Dalgaard TS. Pustulan activates chicken bone marrow-derived dendritic cells in vitro and promotes ex vivo Cd4(+) T cell recall response to infectious bronchitis virus. Vaccines (Basel) (2020) 8(2):226. doi: 10.3390/vaccines8020226 32429204 PMC7349971

[38] Birkner S Weber S Dohle A Schmahl G Föllmann W. Growth and characterisation of primary bovine colon epithelial cells in vitro. Alternatives to Lab Anim ATLA (2004) 32(6):555–71. doi: 10.1177/026119290403200607 15757494

[39] Stein M Keshav S Harris N Gordon S. Interleukin 4 potently enhances murine macrophage mannose receptor activity: A marker of alternative immunologic macrophage activation. J Exp Med (1992) 176(1):287–92. doi: 10.1084/jem.176.1.287 PMC2119288 1613462

[40] Sutton KM Hu T Wu Z Siklodi B Vervelde L Kaiser P. The functions of the avian receptor activator of nf-kappab ligand (Rankl) and its receptors, rank and osteoprotegerin, are evolutionarily conserved. Dev Comp Immunol (2015) 51(1):170–84. doi: 10.1016/j.dci.2015.03.006 25796577

[41] Iwasaki A Medzhitov R. Control of adaptive immunity by the innate immune system. Nat Immunol (2015) 16(4):343–53. doi: 10.1038/ni.3123 PMC4507498 25789684

[42] See P Dutertre C-A Chen J Günther P McGovern N Irac SE. Mapping the human dc lineage through the integration of high-dimensional techniques. Science (2017) 356(6342):eaag3009. doi: 10.1126/science.aag3009 28473638 PMC7611082

[43] Celada A Borràs FE Soler C Lloberas J Klemsz M van Beveren C. The transcription factor pu. 1 is involved in macrophage proliferation. J Exp Med (1996) 184(1):61–9. doi: 10.1084/jem.184.1.61 PMC2192661 8691150

[44] Tanaka T Akira S Yoshida K Umemoto M Yoneda Y Shirafuji N. Targeted disruption of the nf-Il6 gene discloses its essential role in bacteria killing and tumor cytotoxicity by macrophages. Cell (1995) 80(2):353–61. doi: 10.1016/0092-8674(95)90418-2 7530603

[45] Kernbauer E Maier V Stoiber D Strobl B Schneckenleithner C Sexl V. Conditional Stat1 ablation reveals the importance of interferon signaling for immunity to listeria monocytogenes infection. PloS Pathog (2012) 8(6):e1002763. doi: 10.1371/journal.ppat.1002763 22719255 PMC3375314

[46] Delamarre L Pack M Chang H Mellman I Trombetta ESJS. Differential lysosomal proteolysis in antigen-presenting cells determines antigen fate. Science (2005) 307(5715):1630–4. doi: 10.1126/science.1108003 15761154

[47] Dhami R Schuchman EH. Mannose 6-phosphate receptor-mediated uptake is defective in acid sphingomyelinase-deficient macrophages: Implications for niemann-pick disease enzyme replacement therapy. J Biol Chem (2004) 279(2):1526–32. doi: 10.1074/jbc.M309465200 14557264

[48] Olson MC Scott EW Hack AA Su GH Tenen DG Singh H. Pu. 1 is not essential for early myeloid gene expression but is required for terminal myeloid differentiation. Immunity (1995) 3(6):703–14. doi: 10.1016/1074-7613(95)90060-8 8777716

[49] Contreras V Urien C Guiton R Alexandre Y Manh T-PV Andrieu T. Existence of Cd8α-like dendritic cells with a conserved functional specialization and a common molecular signature in distant mammalian species. J Immunol (2010) 185(6):3313–25. doi: 10.4049/jimmunol.1000824 20702727

[50] Summerfield A Auray G Ricklin M. Comparative dendritic cell biology of veterinary mammals. Annu Rev Anim Biosci (2015) 3(1):533–57. doi: 10.1146/annurev-animal-022114-111009 25387110

[51] Karsunky H Merad M Cozzio A Weissman IL Manz MG. Flt3 ligand regulates dendritic cell development from Flt3+ lymphoid and myeloid-committed progenitors to Flt3+ dendritic cells in vivo. J Exp Med (2003) 198(2):305–13. doi: 10.1084/jem.20030323 PMC2194067 12874263

[52] Hildner K Edelson BT Purtha WE Diamond M Matsushita H Kohyama M. Batf3 deficiency reveals a critical role for Cd8alpha+ dendritic cells in cytotoxic T cell immunity. Science (2008) 322(5904):1097–100. doi: 10.1126/science.1164206 PMC2756611 19008445

[53] Crozat K Tamoutounour S Manh T-PV Fossum E Luche H Ardouin L. Cutting edge: Expression of Xcr1 defines mouse lymphoid-tissue resident and migratory dendritic cells of the Cd8α+ type. J Immunol (2011) 187(9):4411–5. doi: 10.4049/jimmunol.1101717 21948982

[54] Satpathy AT Brown RA Gomulia E Briseño CG Mumbach MR Pan Z. Expression of the transcription factor Zbtb46 distinguishes human histiocytic disorders of classical dendritic cell origin. Modern Pathol (2018) 31(9):1479–86. doi: 10.1038/s41379-018-0052-4 PMC6138663 29743654

[55] Carrasco D Bravo RJO. Tissue-specific expression of the fos-related transcription factor fra-2 during mouse development. Oncogene (1995) 10(6):1069–79. 7700631

[56] Sabatakos G Sims N Chen J Aoki K Kelz M Amling M. Overexpression of Δfosb transcription factor (S) increases bone formation and inhibits adipogenesis. Nat Med (2000) 6(9):985–90. doi: 10.1038/79683 10973317

[57] Fleetwood AJ Lawrence T Hamilton JA. Granulocyte-macrophage colony-stimulating factor (Csf) and macrophage csf-dependent macrophage phenotypes display differences in cytokine profiles and transcription factor activities: Implications for csf blockade in inflammation. J Immunol (2007) 178(8):5245–52. doi: 10.4049/jimmunol.178.8.5245 17404308

[58] Larsen AMH Kuczek DE Kalvisa A Siersbaek MS Thorseth ML Johansen AZ. Collagen density modulates the immunosuppressive functions of macrophages. J Immunol (2020) 205(5):1461–72. doi: 10.4049/jimmunol.1900789 32839214

[59] Rodríguez-Prados J-C Través PG Cuenca J Rico D Aragonés J Martín-Sanz P. Substrate fate in activated macrophages: A comparison between innate, classic, and alternative activation. J Immunol (2010) 185(1):605–14. doi: 10.4049/jimmunol.0901698 20498354

[60] Li F Okreglicka KM Pohlmeier LM Schneider C Kopf M. Fetal monocytes possess increased metabolic capacity and replace primitive macrophages in tissue macrophage development. EMBO J (2020) 39(3):e103205. doi: 10.15252/embj.2019103205 31894879 PMC6996567

[61] Young R Bush SJ Lefevre L McCulloch MEB Lisowski ZM Muriuki C. Species-specific transcriptional regulation of genes involved in nitric oxide production and arginine metabolism in macrophages. Immuno Horizons (2018) 2(1):27–37. doi: 10.4049/immunohorizons.1700073 PMC6245571 30467554

[62] Gordon S Martinez FO. Alternative activation of macrophages: Mechanism and functions. Immunity (2010) 32(5):593–604. doi: 10.1016/j.immuni.2010.05.007 20510870

[63] Mosser DM Edwards JP. Exploring the full spectrum of macrophage activation. Nat Rev Immunol (2008) 8(12):958–69. doi: 10.1038/nri2448 PMC2724991 19029990

[64] Orecchioni M Ghosheh Y Pramod AB Ley KJ. Macrophage polarization: Different gene signatures in M1 (Lps+) vs. classically and M2 (Lps–) vs. alternatively activated macrophages. Front Immunol (2019) 10:1084. doi: 10.3389/fimmu.2019.01084 31178859 PMC6543837

[65] Liao X Sharma N Kapadia F Zhou G Lu Y Hong H. Krüppel-like factor 4 regulates macrophage polarization. JCI (2011) 121(7):2736–49. doi: 10.1172/JCI45444 PMC3223832 21670502

[66] Schnoor M Cullen P Lorkowski J Stolle K Robenek H Troyer D. Production of type vi collagen by human macrophages: A new dimension in macrophage functional heterogeneity. J Immunol (2008) 180(8):5707–19. doi: 10.4049/jimmunol.180.8.5707 18390756

[67] Erlich Z Shlomovitz I Edry-Botzer L Cohen H Frank D Wang H. Macrophages, rather than dcs, are responsible for inflammasome activity in the gm-csf bmdc model. Nat Immunol (2019) 20(4):397–406. doi: 10.1038/s41590-019-0313-5 30742078

