# Supplementary material for: Chicken CSF2 and IL-4-, and CSF2-dependent bone marrow cultures differentiate into macrophages over time
Source: Front Immunol. 2022 Dec 21;13:1064084. doi: 10.3389/fimmu.2022.1064084 (PMC9812659; doi:10.3389/fimmu.2022.1064084)
Supplement: Supplementary file 1 [file Presentation_1.pptx]

## Slide 1
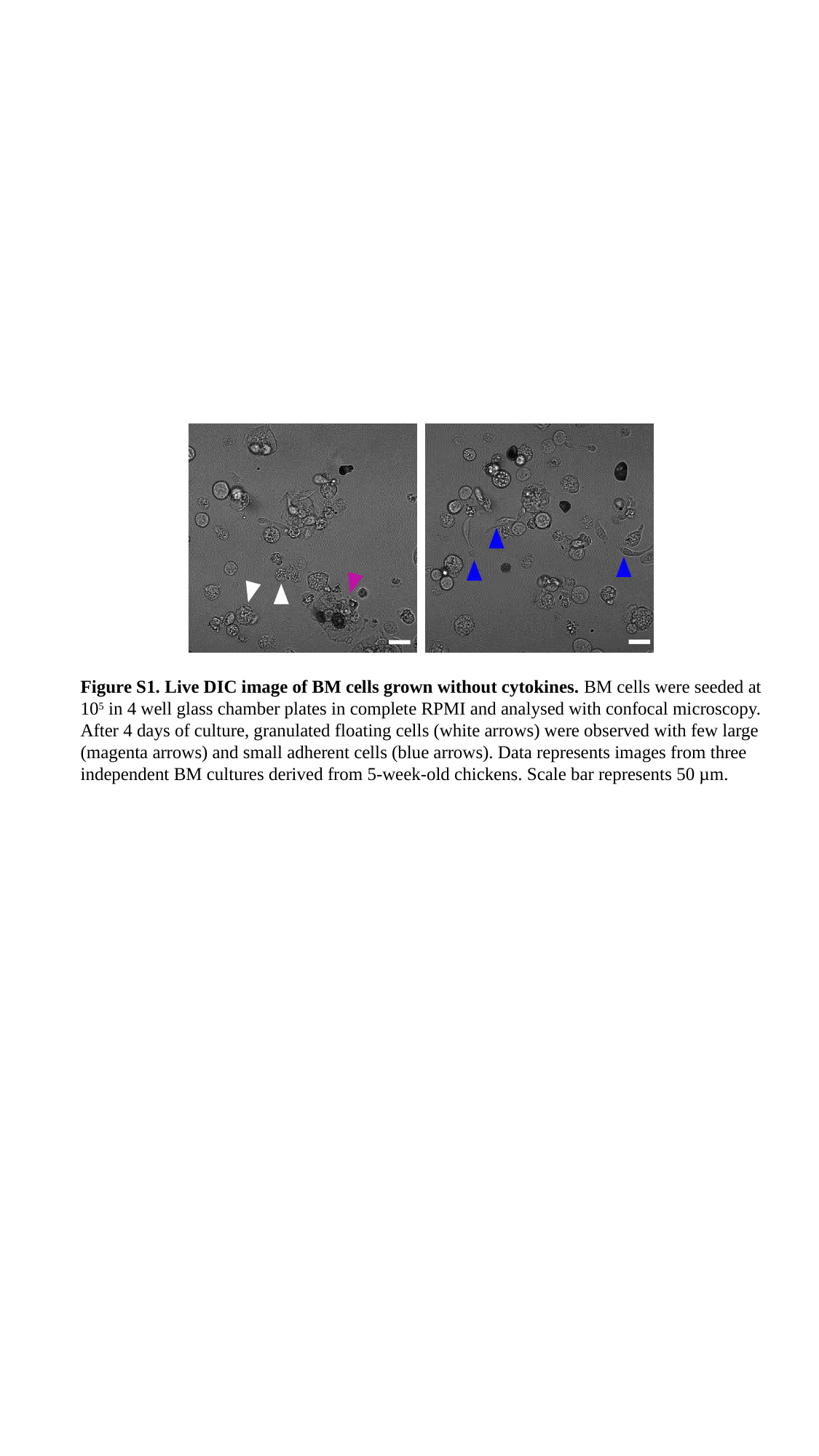

Figure S1. Live DIC image of BM cells grown without cytokines. BM cells were seeded at 105 in 4 well glass chamber plates in complete RPMI and analysed with confocal microscopy. After 4 days of culture, granulated floating cells (white arrows) were observed with few large (magenta arrows) and small adherent cells (blue arrows). Data represents images from three independent BM cultures derived from 5-week-old chickens. Scale bar represents 50 µm.

## Slide 2
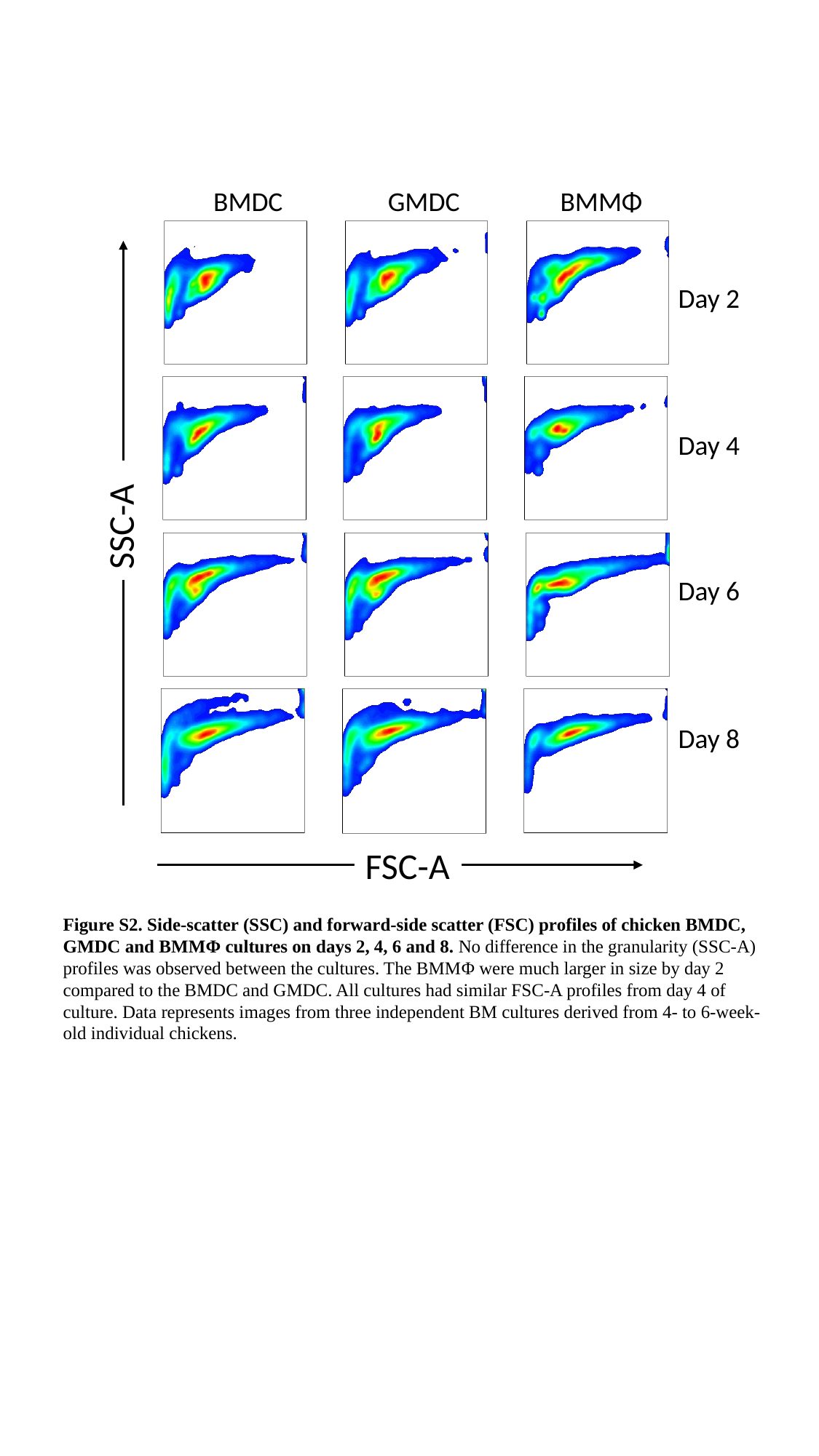

BMDC
GMDC
BMMΦ
Day 2
Day 4
SSC-A
Day 6
Day 8
FSC-A
Figure S2. Side-scatter (SSC) and forward-side scatter (FSC) profiles of chicken BMDC, GMDC and BMMΦ cultures on days 2, 4, 6 and 8. No difference in the granularity (SSC-A) profiles was observed between the cultures. The BMMΦ were much larger in size by day 2 compared to the BMDC and GMDC. All cultures had similar FSC-A profiles from day 4 of culture. Data represents images from three independent BM cultures derived from 4- to 6-week-old individual chickens.

## Slide 3
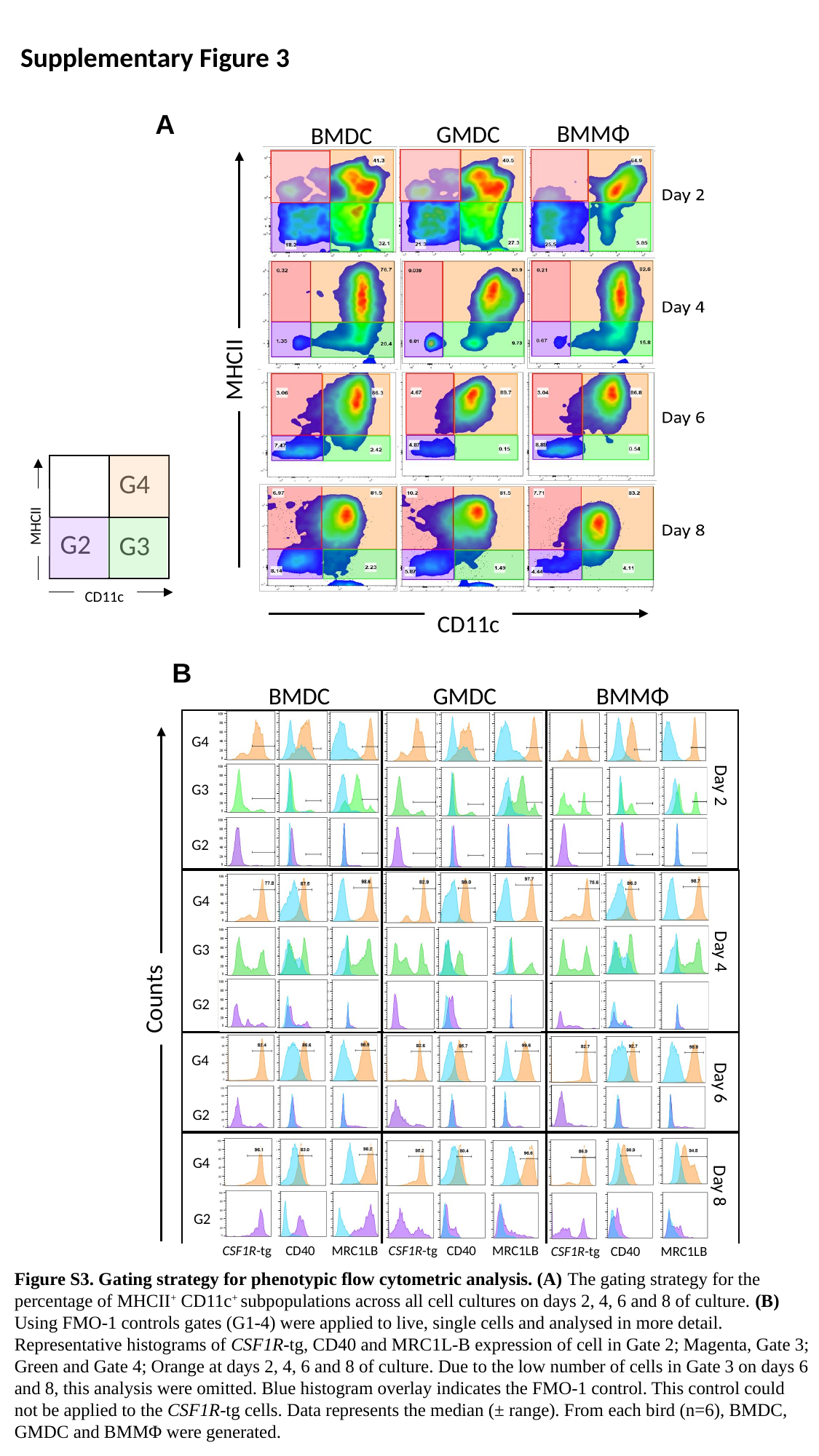

Supplementary Figure 3
A
BMMΦ
GMDC
BMDC
MHCII
CD11c
G4
G2
G3
MHCII
CD11c
B
BMDC
GMDC
BMMΦ
G4
Day 2
G3
G2
G4
G3
Day 4
Counts
G2
G4
Day 6
G2
G4
Day 8
G2
CSF1R-tg
CD40
MRC1LB
CSF1R-tg
CD40
MRC1LB
CSF1R-tg
CD40
MRC1LB
Figure S3. Gating strategy for phenotypic flow cytometric analysis. (A) The gating strategy for the percentage of MHCII+ CD11c+ subpopulations across all cell cultures on days 2, 4, 6 and 8 of culture. (B) Using FMO-1 controls gates (G1-4) were applied to live, single cells and analysed in more detail. Representative histograms of CSF1R-tg, CD40 and MRC1L-B expression of cell in Gate 2; Magenta, Gate 3; Green and Gate 4; Orange at days 2, 4, 6 and 8 of culture. Due to the low number of cells in Gate 3 on days 6 and 8, this analysis were omitted. Blue histogram overlay indicates the FMO-1 control. This control could not be applied to the CSF1R-tg cells. Data represents the median (± range). From each bird (n=6), BMDC, GMDC and BMMΦ were generated.
